# Geographical Origin Authentication of Leaves and Drupes from *Olea europaea* via ^1^H NMR and Excitation–Emission Fluorescence Spectroscopy: A Data Fusion Approach

**DOI:** 10.3390/molecules30153208

**Published:** 2025-07-30

**Authors:** Duccio Tatini, Flavia Bisozzi, Sara Costantini, Giacomo Fattori, Amedeo Boldrini, Michele Baglioni, Claudia Bonechi, Alessandro Donati, Cristiana Tozzi, Angelo Riccaboni, Gabriella Tamasi, Claudio Rossi

**Affiliations:** 1 Department of Biotechnology, Chemistry and Pharmacy, University of Siena, Via Aldo Moro 2, 53100 Siena, Italy; flavia.bisozzi@student.unisi.it (F.B.); sara.costantini@student.unisi.it (S.C.); giacomo.fattori@student.unisi.it (G.F.); amedeo.boldrini@student.unisi.it (A.B.); claudia.bonechi@unisi.it (C.B.); alessandro.donati@unisi.it (A.D.); gabriella.tamasi@unisi.it (G.T.); claudio.rossi@unisi.it (C.R.); 2 Centre for Colloid and Surface Science (CSGI), University of Florence, Via della Lastruccia 3, 50019 Sesto Fiorentino, Italy; 3 Santa Chiara Lab, University of Siena, Via Valdimontone 1, 53100 Siena, Italy; cristiana.tozzi@unisi.it (C.T.); angelo.riccaboni@unisi.it (A.R.)

**Keywords:** chemometrics, machine learning, nuclear magnetic resonance, fluorescence, olive, drupes, leaves, SIMCA, PARAFAC, ComDim, multiblock

## Abstract

Geographical origin authentication of agrifood products is essential for ensuring their quality, preventing fraud, and maintaining consumers’ trust. In this study, we used proton nuclear magnetic resonance (^1^H NMR) and excitation–emission matrix (EEM) fluorescence spectroscopy combined with chemometric methods for the geographical origin characterization of olive drupes and leaves from different Tuscany subregions, where olive oil production is relevant. Single-block approaches were implemented for individual datasets, using principal component analysis (PCA) for data visualization and Soft Independent Modeling of Class Analogy (SIMCA) for sample classification. ^1^H NMR spectroscopy provided detailed metabolomic profiles, identifying key compounds such as polyphenols and organic acids that contribute to geographical differentiation. EEM fluorescence spectroscopy, in combination with Parallel Factor Analysis (PARAFAC), revealed distinctive fluorescence signatures associated with polyphenolic content. A mid-level data fusion strategy, integrating the common dimensions (ComDim) method, was explored to improve the models’ performance. The results demonstrated that both spectroscopic techniques independently provided valuable insights in terms of geographical characterization, while data fusion further improved the model performances, particularly for olive drupes. Notably, this study represents the first attempt to apply EEM fluorescence for the geographical classification of olive drupes and leaves, highlighting its potential as a complementary tool in geographic origin authentication. The integration of advanced spectroscopic and chemometric methods offers a reliable approach for the differentiation of samples from closely related areas at a subregional level.

## 1. Introduction

The global market for olive oil and its related products is expanding rapidly, driven by consumer preferences for healthy, high-quality foods [1,2]. Among the various Italian regions known for olive oil production, Tuscany stands out for its premium products, which are highly valued for their unique sensory attributes and quality [3]. However, with increasing market demand, the challenge of ensuring the authenticity of these products becomes of pivotal importance, since fraudulent practices such as mislabeling and adulteration are frequently encountered [4,5]. Thus, robust and reliable methods for assessing the geographical origin of olive oils are essential to maintain consumer trust and protect the integrity of this regional specialty. To this aim, understanding the chemical composition of olive drupes and leaves is a crucial step before investigating the final extra virgin olive oil product [6]. Since the chemical composition of these plant matrices directly depends on environmental factors such as soil composition, climate, and agricultural practices, they provide a more direct connection to the geographical origin [7,8,9,10]. The characterization of their metabolic profile allows for the identification of region-specific markers that influence the composition of the extracted oil, improving its traceability and authenticity [11].

Several analytical procedures have been proposed for olive oil, drupes, and leaves characterization, including mass spectrometry, gas and liquid chromatography, as well as different spectroscopic techniques, often combined with statistical and chemometrics approaches [9,12,13,14,15,16,17,18]. Among these, nuclear magnetic resonance (NMR) spectroscopy, particularly ^1^H NMR, has emerged as a powerful and reliable analytical technique for the geographical authentication of olive oils, and for the varietal and metabolomic analysis of olive drupes and leaves [19,20,21]. Three main methodologies have been explored in ^1^H NMR and, more in general, in metabolomic investigations like mass spectrometry: target analysis, metabolic profiling, and metabolic fingerprinting [22]. Through target analysis, a specific metabolite or a class of metabolites present in olive oil is detected and quantified, usually after a selective extraction. In the metabolic profiling, different selected metabolites from various classes of compounds are identified, usually without any selective isolation. NMR metabolic fingerprinting is often performed on the full ^1^H spectral data, excluding any a priori selection of specific metabolites. Both metabolic profiling and fingerprinting enable the classification of olive oil samples based on factors such as geographic origin, variety, harvest timing, and aging, by combining the analysis of the spectral features with multivariate statistics and chemometrics [23]. An overview of the recent progresses in the authentication of olive oils and related vegetable matrixes via ^1^H NMR and chemometric methods can be found in the works by Fanizzi et al. [24], Camin et al. [25], Beteinakis et al. [20], Dais et al. [21], and references therein. The high reproducibility and non-destructive nature make ^1^H NMR particularly suitable for the differentiation of products from distinct geographical regions in routine analysis [26,27].

In recent years, excitation–emission matrix (EEM) fluorescence spectroscopy has attracted increasing attention for its ability to identify and characterize fluorescent compounds in food matrices [28]. Several studies reported on the application of EEM fluorescence spectroscopy for authentication and classification purposes in wines [29], spirits [30], edible oils [31], fruits [32], cereals [33], and dairy products [34,35]. In the case of olive oils, this technique is particularly effective in detecting phenolic compounds, tocopherols, pigments, and other secondary metabolites like oxidation products that contribute to the oil’s quality and authenticity [36]. However, to the best of our knowledge, EEM fluorescence spectroscopy has not previously been applied specifically for the purpose of determining the geographical origin of olive drupes and leaves. Moreover, this technique presents several advantages, including its rapid, non-destructive nature and high sensitivity, without requiring extensive sample preparation or expensive instrumentation [28]. Within the three-dimensional structures of EEM data, it is possible to detect variations in excitation and emission wavelengths and capture the complex fluorescence behavior of multiple compounds simultaneously. Nevertheless, the complexity of EEM datasets requires specific chemometric approaches to extract meaningful information. Multitensor decomposition methods, and, in particular, Parallel Factor Analysis (PARAFAC), have proven to be highly effective for resolving overlapping fluorescence signals [37,38]. PARAFAC decomposes EEM datasets into individual components representing chemically meaningful fluorophores, facilitating the isolation and identification of key compounds that contribute to geographic origin and product quality authentication. An alternative strategy for the classification of EEM data involves reshaping the 3D array into a 2D matrix, where each sample’s EEM is converted into a row vector [39]. This unfolding procedure allows the application of first-order visualization and classification algorithms, such as principal component analysis (PCA), discriminant analysis (DA) or class-modeling algorithms like Soft Independent Modeling of Class Analogy (SIMCA) [40,41,42].

Despite the high molecular specificity and reproducibility of ^1^H NMR, its implementation in routine industrial workflows is limited by practical constraints, including high acquisition and maintenance costs. In contrast, more accessible techniques such as NIR, FTIR, fluorescence, and UV-Vis spectroscopies are widely adopted in routine analysis of agrifood products [43,44,45] due to their lower operational costs, minimal sample preparation, and the availability of portable instrumentation. However, these methods often suffer from lower selectivity and resolution, particularly in non-targeted authentication. In this context, high-resolution techniques like ^1^H NMR can play a key role in confirmatory and in-depth analyses in centralized laboratories supporting regulatory control. Moreover, integrating ^1^H NMR with complementary and portable methods via data fusion may offer a practical compromise between analytical performance and field applicability.

The present study focuses on the geographical origin authentication of olive drupes and leaves from the 2022 harvest in Tuscany, using a comprehensive strategy combining ^1^H NMR, EEM fluorescence spectroscopy, and multivariate analysis. The objective was to integrate an inexpensive and relatively simple technique (EEM fluorescence) together with NMR via data fusion to enhance the sample classification at a subregional level.

First, the individual datasets from the two spectroscopic techniques are analyzed independently, and PCA is applied to detect potential clustering patterns among the samples according to their geographical origin. Additionally, SIMCA was implemented to develop a classification model for samples originating from a specific subregional area.

Then, a multi-block approach is explored to improve the sample differentiation through data fusion. Data fusion strategies are emerging as a promising tool for food quality assessment and geographical origin characterization, and they can be categorized into low-level, mid-level, and high-level [46]. In this study, a mid-level data fusion approach, which implements the common dimensions (ComDim) algorithm, is applied. This method extracts shared patterns between ^1^H NMR and EEM fluorescence blocks by identifying common dimensions (CDs) that summarize the variance-covariance structure of the combined data [47]. This approach offers the advantage of balancing the individual contributions of each dataset while maintaining interpretability of the results. The ComDim algorithm facilitates the identification of underlying trends and relationships that might not be apparent when analyzing each dataset independently. To the best of our knowledge, only one paper has reported on the application of ComDim for olive oil characterization, albeit using different analytical techniques (UV–Vis spectroscopy, Near-Infrared spectroscopy and gas chromatography) [48]; in the case of drupes and leaves this is the first study so far.

To further improve the classification performance and evaluate the benefits of data integration, a SIMCA-based one-class modeling strategy is implemented using the multiblock ComDim outputs, and its performance was systematically compared to SIMCA models developed on individual datasets.

In this perspective, this study aims to develop and validate a reliable method for the geographical origin authentication of olive vegetal matrices, starting from spectroscopic data. The findings contribute to the expanding research field on the authentication of olive-derived products, demonstrating the potential of spectroscopy and chemometrics integration to support traceability and quality assessment efforts across the production chain.

## 2. Results and Discussion

Fifty-one drupe and thirty-one leaf samples harvested in September and October 2022 from different geographical areas of Tuscany (Chianti–Siena, Grosseto, and Val d’Orcia) were collected and analyzed.

**Figure 1:**
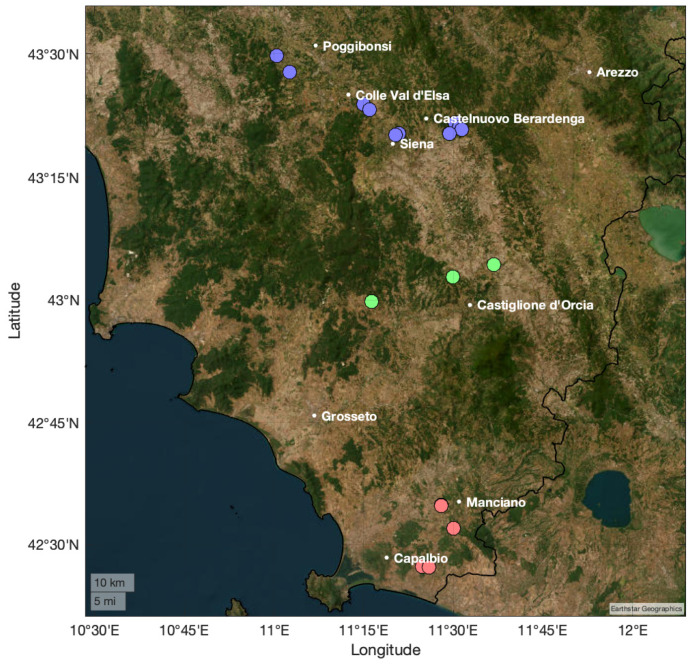
Sampling sites for leaves and drupes. The three different selected regions are evidenced by colored markers as follows: (blue) Chianti and Siena; (green) Val d’Orcia; (red) Grosseto.

These subregions were selected according to the Protected Designation of Origin (PDO) classification and to the chemical composition of the soil from previous studies [6,49]. The sampling sites are shown in Figure 1, and the detailed information about the samples is reported in Table S1 in the Supplementary Material file, along with environmental and climatic metadata.

The results obtained from ^1^H NMR and EEM fluorescence experiments for the olive leaves and drupes are first examined separately to highlight the specific contributions of each technique. Then, the outcomes of the multiblock analysis on the merged datasets based on the ComDim approach are discussed.

### 2.1. Olive Leaves—^1^H NMR and EEM Fluorescence Spectroscopy—Single-Technique Approach

**Figure 2:**
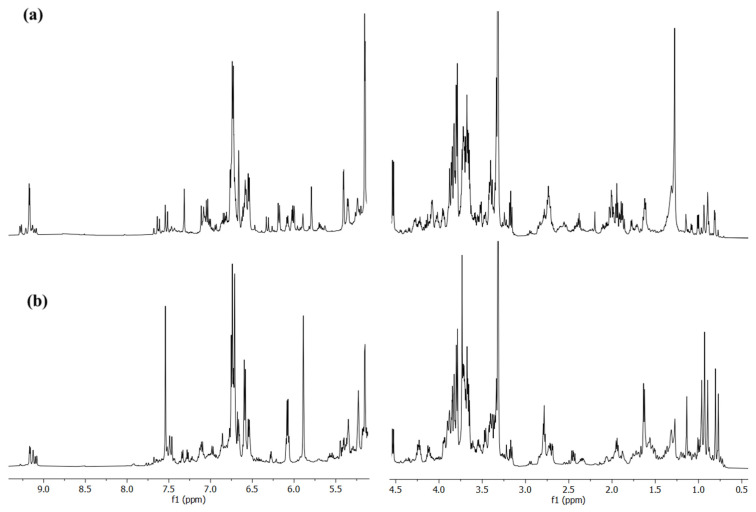
^1^H NMR spectra recorded for a single olive leaves’ (**a**) and drupes’ (**b**) sample from Siena region, which are representative of the typical NMR profile of leaf and drupe extracts.

The ^1^H NMR spectrum of a sample from the Chianti–Siena region is reported in Figure 2a, which represents the typical NMR profile of olive leaves’ extract. The visual appearance of such spectra is quite complex, due to the presence of several overlapping signals that can be ascribed to different classes of compounds like secoiridoids (oleuropein, ligstroside, oleocanthal, and oleacin), sugars, other polyphenols (tyrosol and hydroxytyrosol), and organic acids. The detailed chemical shifts assignment is listed in Table S2 in the Supplementary Material.

The most intense signals are located in the 3–5.5 ppm region, and they can be primarily assigned to sugars and sugar alcohols like mannitol, glucose, and sucrose, which are the most abundant soluble sugars in olive leaves [50]. Specifically, the doublets centered at 5.13 ppm and 4.52 ppm are identified as the anomeric protons of α- and β-glucose, respectively. An additional characteristic doublet at 5.39 ppm, attributed to sucrose, is also detected [51]. Mannitol exhibits multiple signal patterns in the range 3.65–3.90 ppm [52]. Several intense NMR signals can be observed between 5.80 and 7.52 ppm and they can be attributed to oleuropein, one of the main constituent of olive leaf extracts [53]: the presence of this glycosylated secoiridoid is confirmed by additional characteristic resonances in the 1.61–4.16 ppm region (for further details see Table S2 in the Supplementary Material) [54,55]. These signals originating from oleuropein could be potentially superimposed to the signals of ligstroside, another secoiridoid that differs from oleuropein by one hydroxyl group. Nevertheless, a recent work evidenced that in the extracts of the same olive leaves’ varieties the concentration of oleuropein is one order of magnitude higher than ligstroside [9]. Signals in the 9.0–9.2 ppm range are attributed to the aldehydic proton of oleocanthal and oleacin, alongside their hemiacetal derivatives [56]. These compounds originated from the demethylation and spontaneous decarboxylation of the aglycone form of ligstroside and oleuropein, respectively [57]. A characteristic singlet at 8.48 ppm, attributed to formate, can also be detected. In the 6.22–7.52 ppm range, NMR signals corresponding to phenyl alcohol moieties of hydroxytyrosol are observed, along with different characteristic resonances of luteolin [55,58]. In the aliphatic region organic acids, including malic (2.29–2.70 ppm), citric (2.50–2.70 ppm), succinic (2.40 ppm), quinic (1.80–2.09 ppm), and lactic (1.32 ppm) acids are also identified [51,52,59]. Signals between 0.70 and 1.13 ppm can be attributed to maslinic and oleanolic acids [60].

**Figure 3:**
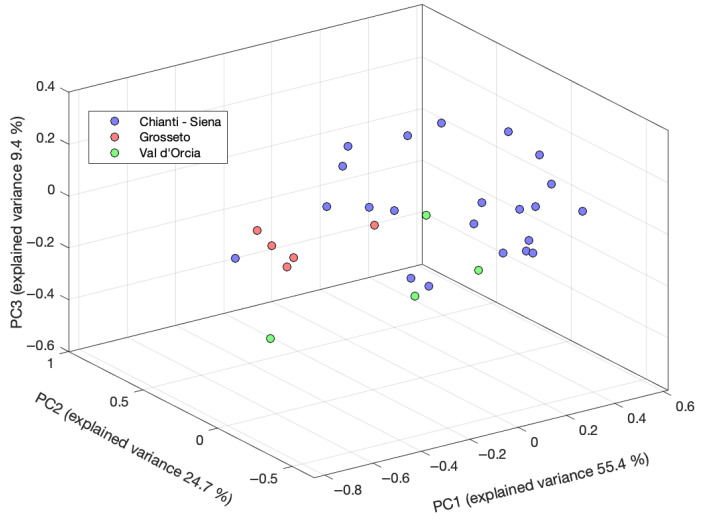
Three-dimensional PCA score plot for ^1^H NMR data of the olive leaf samples.

First, an exploratory data analysis was performed via PCA on the NMR leaves dataset. The resulting score plot for the first three principal components, accounting for 89.5% of the original variance, is reported in Figure 3. The loading plot, along with the 2D score plots for PC1, PC2, and PC3, is reported in Figure S1 of the Supplementary Material.

The distribution of the samples along the PCs shows a partial differentiation according to the different geographical origins. The samples from Chianti–Siena region are relatively well clustered, similarly to the samples from Grosseto, which form a separate, though more scattered, group. The samples from Val d’Orcia are more widely distributed, showing a significant overlap with the other regions. The separation observed along PC1 accounts for the most significant differentiation among the groups, particularly between Chianti–Siena and the other regions. Notably, this trend seems to correlate with the latitude of the sampling sites, as evidenced in the 2D score plots in Figure S1 (Supplementary Material). The samples from Chianti–Siena are located at positive PC1 values, which gradually shift toward more negative values when moving from Val d’Orcia to Grosseto, so from central to southern Tuscany.

The analysis of the PCA loadings (Figure S1 in the Supplementary Material) reveals that the major contributions in terms of individual variables (i.e., buckets) are given by the oleuropein/ligstroside signals at 1.64, 2.80, 3.71 and 6.73 ppm, followed by mannitol (3.65, 3.81), glucose (3.37, 4.53), and quinic acid (1.96). These findings are in good agreement with the results obtained from the analysis of the phenolic composition via high-performance liquid chromatography–high-resolution mass spectrometry (HPLC-HRMS) on the same dataset, identifying ligstroside as one of the major contributors to geographic discrimination [9].

Considering the results obtained from the explorative PCA, SIMCA was implemented to build a classification model for the Chianti–Siena subregional area. Among the three geographical areas considered, the samples from this subregion exhibit a relatively more compact and coherent distribution despite the overall absence of clear global separation. This apparent in-class consistency provides a suitable foundation for a class-modeling approach, aiming to distinguish Chianti–Siena samples from those belonging to other regions.

The model performance parameters are listed in Table 1, while the SIMCA distance plot is reported in the Supplementary Material (Figure S2).

The SIMCA model shows good performance in classifying samples from the Chianti–Siena subregion in both the training and test sets, achieving a prediction accuracy of 83%, albeit with relatively low specificity. The analysis of the model loadings supports the findings from the PCA, highlighting the predominant contribution of oleuropein/ligstroside (δ 1.64, 2.80, 3.71 ppm) and sugars, specifically mannitol (δ 3.65, 3.81 ppm) and glucose (δ 3.37, 4.53 ppm).

**Figure 4:**
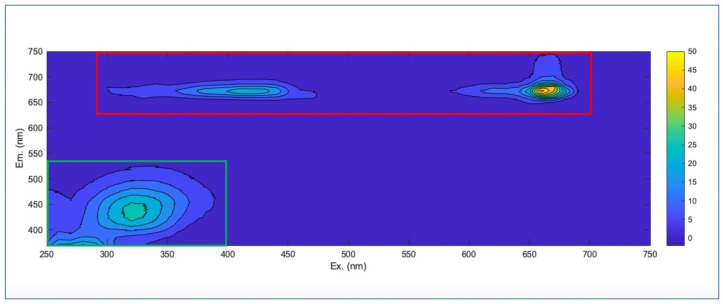
Processed full excitation–emission fluorescence map of olive leaves. The green area represents the chlorophylls’ region (Region A), and the red area corresponds to the emission region of cellular fluorophores like amino acids, phenolic compounds, vitamins, and organic polymers (Region B).

Figure 4 shows the full excitation–emission landscape recorded for a single representative olive leaf sample from the Chianti–Siena region. Two main fluorescence emission regions can be detected: the first one (Region A) presents excitation and emission wavelengths in the range 300–700 and 650–750 nm, respectively, while the second area (Region B) has excitation wavelengths from 250 to 400 nm and emission wavelengths from 370 to 530 nm.

According to the existing literature, in olive oils [61,62,63], fruit samples [28,32] and grapevine leaf extracts [64], the fluorescence signals in Region A are usually ascribed to the emission of chlorophylls and pheophytins, while in Region B several fluorescent compounds may emit, like polyphenols, vitamins, amino acids, and organic polymers. To the best of our knowledge, this is the first work reporting the characterization of olive leaves’ emission profiles via EEM fluorescence spectroscopy.

Since chlorophylls may affect the performance and reliability of the models because of their high fluorescence intensity and high variability (mainly due to different cultivars and climatic conditions) the two emission regions were analyzed separately [65,66].

The excitation and emission loadings’ profiles obtained through PARAFAC decomposition for Region A and B are shown in Figures S3 and S4 in the Supplementary Material, while the assignment of fluorescent compounds corresponding to the emission patterns in olive leaf samples is summarized in Table 2.

For Region A, a three-component PARAFAC model was found to produce the most accurate results: the three different contributions can be ascribed to the presence of chlorophyll *a*, chlorophyll *b*, and pheophytin *a*, respectively, in agreement with previous works [64,67]. For Region B, the optimal number of components was three: the first one exhibits a well-defined band with an excitation maximum at 320 nm and an emission maximum at 435 nm, which can be related to the presence of chlorogenic acid and related compounds [64,68]. The second component shows a single excitation maximum at 280 nm, while the emission band is only partially visible since the maximum is located below 370 nm, outside the investigated wavelength range. Nevertheless, this component can be assigned to the fluorescence profile of phenolic compounds, as extensively reported in the case of olive oil [69,70,71]. For the third component, the excitation and emission maxima occur at 360 and 465 nm, respectively, and these bands are commonly associated with the presence of tocopherols and tocotrienols [32,62,71].

The concentration mode (score plots) of the PARAFAC model is reported in Figures S3 and S4 in the Supplementary Material to show the relative concentrations of the different classes of fluorophores for each sample. For Region A, the concentration of the first two components, which corresponds to chlorophyll *a* and pheophytin *a*, is higher than pheophytin *b*, despite major fluctuations across the dataset. For Region B, the most abundant fluorescent component is chlorogenic acid, followed by phenolic compounds and tocopherols, respectively.

For geographical authentication purposes, the chlorophyll region (Region A) was excluded from the PCA and SIMCA analysis, since the chlorophylls’ content and the consequent fluorescent emission profile can be remarkably affected by different climatic conditions [40].

The PCA exploratory analysis performed on the EEM data from Region B (Figures S5 and S6 in the Supplementary Material) indicates a limited separation among the three regions: while the samples from Chianti–Siena show a partial clustering, a considerable overlap with Grosseto and Val d’Orcia is observed.

The loading vectors were refolded into EEM-like tridimensional data to identify the regions of the EEM that give the major contributions in the PCA model. The analysis of the refolded loadings revealed that the most relevant excitation–emission wavelength couples are centered around 280/370 nm, corresponding to the fluorescence profile of polyphenols. This result provides confirmation about the importance of these compounds as markers for geographical authentication, as reported in previous studies [6,9]. A secondary contribution is given by excitation–emission signals at 330/435 nm (chlorogenic acid), while tocopherols and tocotrienols do not provide significant contributions.

The classification results obtained from the SIMCA model on Region B are summarized in Table 1, while the distance plot is reported in Figure S7 in the Supplementary Material.

SIMCA results show a predictive performance in the test set comparable to that of the ^1^H NMR model, although slightly lower performance metrics are observed in the calibration set. In particular, the SIMCA model can effectively discriminate between the Chianti–Siena and Val d’Orcia areas, as evidenced by the sharp differences in the distance plot. The loadings analysis confirms the results obtained via PCA, indicating polyphenols and chlorogenic acid fluorescence emissions as the most relevant contributions for the geographical differentiation.

### 2.2. Drupes—^1^H NMR and EEM Fluorescence Spectroscopy—Single-Technique Approach

As in the case of olive leaves, the drupe extracts show a very complex NMR profile due to the presence of multiple metabolites with similar or overlapping signals. The ^1^H NMR spectrum of a sample from the Chianti–Siena region reported in Figure 2b is a good example of a typical olive drupes’ extract. The detailed chemical shifts assignment is listed in Table S3 in the Supplementary Material.

The ^1^H NMR profiles of the drupe extracts share some similarities with the spectra obtained from the leaves: several intense signals related to sugars (glucose, sucrose, and mannitol) are clearly observable between 3 and 4.5 ppm, while the characteristic peaks of oleuropein are detected at 7.52, 6.70, 5.90–6.00, 2.70, and 1.61 ppm. In the range between 5 and 8 ppm the most intense signals are attributed to the presence of verbascoside, quercetin and luteolin [20,72,73,74], while in the aliphatic region the characteristic resonances of maslinic and oleanolic acid [60,75] (0.77–1.13 ppm) along with other different organic acids as lactic (1.32 ppm), acetic (1.85 ppm), quinic (1.86–1.95 ppm), malic (2.31–2.36 ppm), and succinic (2.40 ppm) acids can be detected [51,52,59,76].

Exploratory PCA (Figures S8 and S9 in the Supplementary Material) does not reveal any significant clustering or separation in the sample distribution according to their geographical origin. This can be attributed to the greater chemical complexity of the drupe matrices, which may hinder the ability of unsupervised methods to capture the geographical-related variance. In contrast, as evidenced in the previous section, PCA of leaf extracts (Figure 3) shows a more structured distribution of the samples and a partial differentiation, suggesting a clearer relationship between metabolic composition and the geographic origin.

Subsequently, the ^1^H NMR signals were used as input for SIMCA to build a classification model for the Chianti–Siena area. The performance metrics are listed in Table 1, while the distance plot is reported in the Supplementary Material (Figure S10).

The model shows good overall classification performances, with slightly lower values for the test set. From the comparison with the results obtained for the different vegetable matrices, SIMCA modeling based on ^1^H NMR data seems to provide a more accurate classification for the Chianti–Siena subregion for olive leaves.

The analysis of the loadings obtained from the SIMCA models for the drupe samples evidences that the most relevant contributions in terms of spectral buckets to the geographical classification models are provided by the signals at 1.13 (maslinic and oleanolic acid), 1.31–1.35 (malic acid), 1.80–2.00 (quinic acid), 2.65–2.75 (oleuropein, malic and citric acid), and 3.66–3.82 ppm (mannitol and oleuropein). The comparison with the loading results obtained for leaf samples reveals two noteworthy aspects: firstly, a strong correspondence exists between the metabolites primarily responsible for the classification performance of the models in both leaves and drupes. Secondly, organic acids play a predominant role among these metabolites, alongside specific signals from oleuropein and sugars.

**Figure 5:**
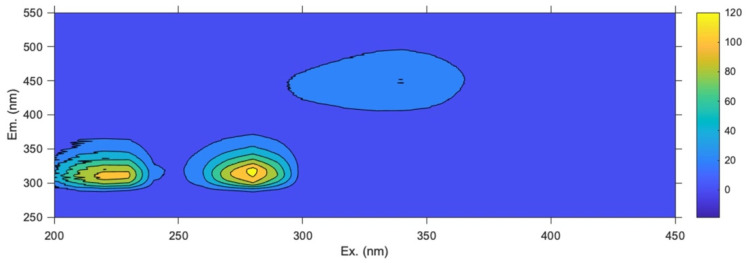
Processed full excitation–emission fluorescence map of olive drupes.

A characteristic fluorescence excitation–emission map for a representative olive drupe extract from the Siena area is reported in Figure 5. Since the emission of chlorophylls and related compounds may negatively affect the chemometric models for geographical origin authentication, the EEM landscapes were acquired by excluding the pigment emission region.

From a visual inspection of the fluorescence maps, three distinct emission areas can be detected, with excitation–emission maxima located at 220/310, 280/315 and 340/450 nm. The PARAFAC modeling confirmed the identification of three major classes of fluorescent components, as shown in Figure S11 in the Supplementary Material. The first component (λ_ex_ = 280 nm; λ_em_ = 315 nm) can be ascribed to the presence of phenolic compounds like catechin and epicatechin [77], while the second component with characteristic λ_ex_ values of 340 nm and λ_em_ of 450 nm, can be associated with tocopherols and tocotrienols, similarly to the case of leaf samples [32,62,71]. The third component shows an intense emission located at 310 nm, with an excitation maximum at 230 nm: these spectral features cannot be unambiguously assigned to specific fluorophores since several phenolic compounds may contribute to the overall emission in this wavelength range [78]. Among these compounds, oleuropein exhibits a well-defined emission band at 310 nm, although in the case of olive oil, the excitation maximum occurs at higher wavelengths (270 nm). The PARAFAC score plot reported in the Supplementary Material in Figure S11 shows that catechin and epicatechin are the most abundant fluorescent compounds across the entire dataset, followed by tocopherols, tocotrienols, and polyphenols/oleuropein.

Exploratory PCA performed on the unfolded EEM fluorescence data of olive drupe extracts (Figures S12 and S13 in the Supplementary Material) reveals a high degree of overlap among the samples from the three geographical regions: a partial clustering along the PC2 can be observed for Val d’Orcia and, to a lesser extent, for Grosseto areas, while the samples from Chianti–Siena region are more scattered. The SIMCA model built for the Chianti–Siena area shows slightly reduced classification performances in both the training and test sets compared to the results obtained with ^1^H NMR data on drupe extracts, as well as to those achieved for the leaf samples (see Table 1 and Figure S14 in the Supplementary Material). This can be attributed to the more complex chemical composition of the drupe’s matrix, which limits the ability of EEM fluorescence spectroscopy to reflect region-specific characteristics.

The examination of the refolded loadings on the principal components demonstrates that the major contributions to the model are provided by the excitation–emission wavelength couples centered at 280/315 nm and 340/450 nm, related to catechin/epicatechin and tocopherols, respectively.

### 2.3. Data Fusion

To further improve the sample differentiation and gain a deeper understanding of the specific contributions provided by the two spectroscopic techniques, the application of a mid-level data fusion based on the ComDim algorithm is explored. As an unsupervised multiblock method, ComDim does not require prior knowledge of the geographical origin of the samples, allowing for an objective integration of complementary information from multiple datasets while preserving the intrinsic data structure.

**Figure 6:**
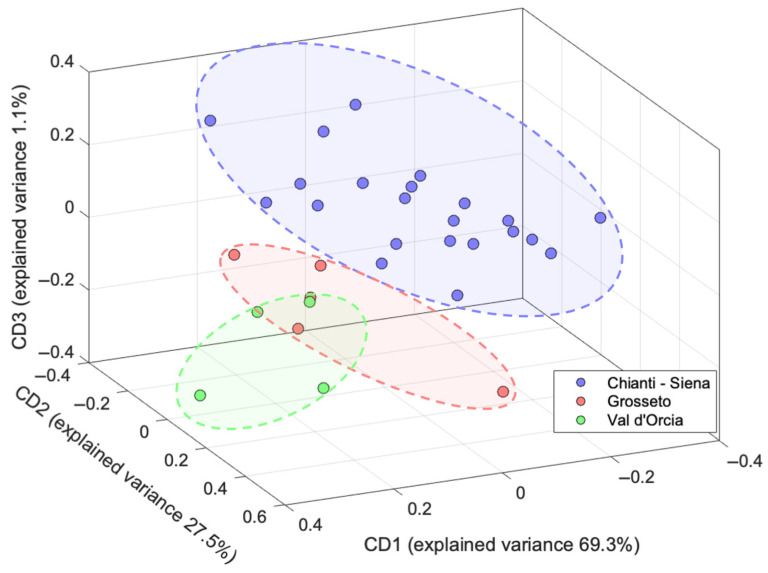
Three-dimensional ComDim score plot for the merged dataset of the olive leaf samples.

As described in the Materials and Methods Section, the individual data blocks from ^1^H NMR and EEM are normalized and concatenated, and then the common dimensions (CD) are extracted. The score plot of the first 3 CDs is reported in Figure 6, while for a comprehensive visualization of all the CDs, see Figure S15 in the Supplementary Material. For each CD, the saliences and loadings are calculated and shown in Figures S15 and S16 in the Supplementary Material.

A 3-CD model is computed from the ComDim analysis, accounting for 97.9% of the total variance. The analysis of the saliences evidences that the ^1^H NMR and EEM blocks provide the major contribution to CD1 and CD2, respectively, while in CD3, the two blocks are almost equivalent.

The implementation of the ComDim algorithm on the merged leaves datasets leads to a clear differentiation of the samples in three distinct clusters, as evidenced in Figure 6. The samples from the Chianti–Siena area are well separated from those belonging to Grosseto-Val d’Orcia: the data fusion approach brings about a significant improvement if compared to the individual techniques, in which PCA shows a partial differentiation with a consistent overlap among the different subregions. Nevertheless, despite the improved clustering, a complete separation between Grosseto and Val d’Orcia cannot be achieved.

A detailed investigation of the loadings (Figure S16 in the Supplementary Material) allows for the identification of the metabolites that are responsible for the geographical origin differentiation. Oleuropein signals at 1.64 and 3.71 ppm, followed by mannitol (3.81 ppm), and oleanolic and maslinic acids (0.90–0.97 ppm) provide the most relevant contributions to the ^1^H NMR block. For the EEM data, the primary contributions arise from phenolic compounds (excitation at 250–280 nm and emission < 370 nm), with a secondary contribution from chlorogenic acid (exc./em. 320/435 nm).

**Figure 7:**
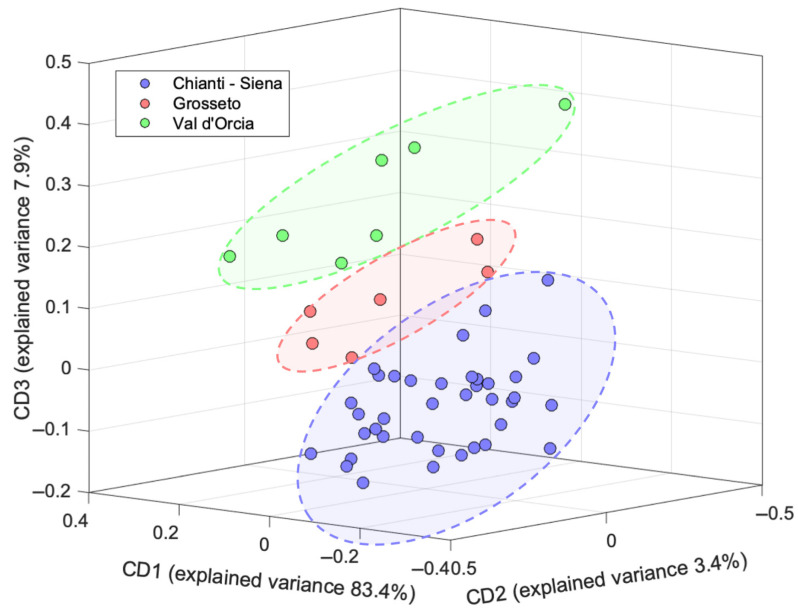
Three-dimensional ComDim score plot for the merged dataset of the olive drupe samples.

A similar mid-level data fusion approach was followed for the investigation of the olive drupe samples. The score plot of the first 3 CDs is reported in Figure 7, while the 2D score plots along with the calculated saliences and loadings are shown in Figures S17 and S18 in the Supplementary Material.

A 3-CD model was computed, accounting for 94.7% of the total variance. The analysis of the saliences (Figure S17 in the Supplementary Material) highlights an almost equal contribution of the two analytical techniques to CD1, while the ^1^H NMR block becomes predominant in CD2 and CD3. The 3D score plot (Figure 7) reveals a significantly improved geographical differentiation compared to the results obtained from the single-block PCA approach. While the unsupervised analysis of the ^1^H NMR and EEM data showed limited or no clear clustering according to geographical origin, the ComDim model highlights a distinct grouping of the samples from Chianti–Siena, Grosseto, and Val d’Orcia.

The investigation of the loadings plots (Figure S18 in the Supplementary Material) evidences that the most relevant contributions in terms of ^1^H NMR signals to the different CDs are provided by the resonances located at 3.67–3.80, 2.72, 1.96, 1.32, and 1.13 ppm, corresponding to mannitol, oleuropein, quinic, lactic, maslinic and oleanolic acids, respectively. For the EEM block, the refolding of the loadings reveals that the most relevant excitation–emission wavelength couples are centered around 340/440 nm, corresponding to the fluorescence profile of tocopherols, while a secondary contribution is given by excitation–emission signals at 230/310 nm, related to phenolic compounds.

Building on the exploratory insights provided by the ComDim score plots, a class-modeling strategy was implemented to assess the ability of the combined spectroscopic information to discriminate the samples from the Chianti–Siena subregion. In particular, a one-class classification model was developed by integrating the ComDim multiblock outputs into a SIMCA-like framework. The model performance metrics are listed in Table 3, while the distance plots for leaves and drupes are reported in Figure S19 in the Supplementary Material.

As the analysis of leaves is concerned, the data-fusion approach did not apport a significant improvement to separate SIMCA models, which already showed good performances, especially the one built on ^1^H NMR data. On the other hand, in the analysis of the geographical origin of drupes, the data-fusion approach allowed for boosting the performance of the models obtainable from the single datasets.

In fact, in terms of accuracy, the multiblock classifier method leads to a significant improvement if compared to EEM-SIMCA, while the performances are similar with respect to the individual ^1^H NMR model. In the test set, the data fusion approach outperforms both the individual models in the training set, achieving the highest overall value of 90%. Sensitivity significantly benefits from data fusion, showing a remarkable increase, particularly in the test set. However, specificity remains modest: this outcome reflects both the intrinsic complexity of the drupe matrix and the challenge of discriminating closely related geographical areas, where similar compositional profiles can limit class separation despite the advantages of multiblock integration.

These results confirm that the integration of ^1^H NMR and fluorescence data can lead to a more robust classification model and improved classification performance compared to single-block approaches, albeit with some limitations. This enhancement is not general but rather matrix-dependent, with clearer benefits observed in the case of olive drupes.

## 3. Materials and Methods

### 3.1. Reagents

Methanol, TSP-D4 (3-(trimethylsilyl)propionic-2,2,3,3-d_4_ acid sodium salt, 98% D), and deuterated solvents (D_2_O, 99.9% D, methanol D4, 99.8% D, H_2_O < 0.03%) were purchased from Merck (Milan, Italy) and used without further purification. Bidistilled water was produced by a Direct Pure UP 10 system (Rephile Bioscience Ltd., Boston, MA, USA).

### 3.2. Sampling and Extraction Protocol

Leaves and olives (three replicates per orchard) were sampled from the four cardinal directions around the perimeter of three different trees at operator height, to ensure a good representation of the internal variability of the sampling site. The total number of samples for leaves and drupes is 31 and 51, respectively. To minimize the effect of drupes’ ripening, the samples were hand-harvested within a one-week period in early October 2022, across geographically close sites in the Tuscany region. Prior to collection, fruits were visually inspected to assess ripening stage, and only healthy, undamaged specimens were selected. Sampling was standardized to trees bearing olives at a consistent phenological stage: approximately half of the fruits were still green, while the other half had begun to transition to the pigmented stage. This ripening phase was chosen to ensure comparability across different cultivars and locations while maintaining the representativeness of typical harvest conditions. These precautions were adopted to reduce the influence of maturity-related changes in metabolite and fluorescence profiles, and to better isolate the geographical contribution in the subsequent chemometric analysis. The samples were stored in plastic bags in the dark until they arrived at the laboratory. Once there, they were washed with ultrapure water, lyophilized at −45 °C and 360 µbar until reaching a constant mass, then blade-milled (Pulverizette 11, Fritsch, Idar-Oberstein, Germany) into a fine powder (500 µm) using a liquid nitrogen bath. The powdered samples were kept frozen and in the dark until analysis. For fluorescence experiments, the leaf and drupe samples were extracted according to a modified version of the International Olive Council’s protocol, as reported in a previous work from our research group [9]. About 500 mg of the dried samples were extracted with 10 mL of an 80:20 methanol/water mixture for 10 min at 25 ± 2 °C in an ultrasonic bath (Sonorex, Bandelin electronic GmbH, Berlin, Germany, operating at 120 W and 35 kHz). The resulting extracts were centrifuged at 3500 rpm for 15 min, and the supernatant was then filtered using 0.22 µm syringe filters. The procedure was repeated three times for a total of 30 mL. Prior to the acquisition of fluorescence maps, the extracts were diluted 1:100 with the 80:20 methanol/water solution. For the ^1^H NMR experiments, a similar protocol was followed, extracting the powdered samples with an 80:20 mixture of deuterated methanol and deuterated water. No additional dilution was required prior to NMR analysis. TSP-d4 (sodium salt of trimethylsilylpropionic acid) was added to each sample as an internal standard (δ = 0) with a final concentration of 0.05% *w*/*v*. All samples and standards were carefully handled to minimize light exposure, and all the experiments were performed in triplicate.

### 3.3. Fluorescence Excitation Emission Matrix (EEM) Experiments

The fluorescence excitation emission matrix (EEM) measurements were performed on an Agilent Cary Eclipse fluorescence spectrophotometer (Agilent Technologies, Milan, Italy) equipped with a xenon flash lamp and a photomultiplier tube as detector. About 3 mL of each sample was placed in a 10 mm quartz cuvette and analyzed at room temperature. The excitation wavelength ranges were 250–750 nm and 200–450 nm for leaves and olive samples, respectively, with 10 nm increments. The emission signals were recorded between 370–750 and 250–550 nm at 1 nm intervals. The excitation and emission ranges were selected on the basis of previous works [40,64] and optimized in order to obtain the best compromise between the inclusion of all the informative fluorescence signals and reasonable acquisition times. The slits of excitation and emission monochromators were set at 5 nm, while the scan rate was set to 600 nm/min. A blank EEM was recorded (80:20 methanol/water solution) and then subtracted from all the fluorescence excitation–emission matrices. The fluorescence excitation–emission matrices were arranged in cubic structures with dimensions of *samples* × *emission wavelength* × *excitation wavelength*. The spectra were preprocessed by removing the Rayleigh and Raman scatter (both first and second order) and interpolating the missing values according to Murphy et al. [37]. The data were normalized to unit variance in sample mode. An exploratory analysis via Parallel Factor Analysis (PARAFAC) decomposition was performed in order to resolve and identify the underlying fluorescent components. For the geographical origin characterization, the cubic structures were unfolded by combining the excitation and emission modes, resulting in 2-D matrices with dimensions of *samples* × (*emission wavelength*…*excitation wavelength*). These unfolded matrices were used as input for the subsequent chemometric analysis.

All the calculations were performed in a MATLAB environment (MATLAB R2023b version, The MathWorks Inc., Natick, MA, USA) using the N-way toolbox [79] and drEEM toolbox [37].

### 3.4. ^1^H-NMR Spectroscopy

For each sample, 1 mL of the extract was placed into a 5 mm NMR test tube. The ^1^H-NMR spectra were recorded on a Bruker DRX-600 AVANCE spectrometer, equipped with an xyz gradient unit and operating at 600.13 MHz. Spectra were processed using Bruker TopSpin software (version 3.6.1, Bruker, Bremen, Germany). The spectra obtained by the Fourier transformation of the free induction decay (FID) were manually phased, and the chemical shifts were reported with respect to the TSP’s signal set at 0 ppm.

The FIDs, relative to the ^1^H NMR experiments, were processed by using NMRProcFlow software, 1.4 version (nmrprocflow.org, INRA UMR 1332 BFP, Bordeaux Metabolomics Facility, Bordeaux, France) [80]. The spectra were phase- and baseline-corrected manually, and sectioned into regular intervals (0.04 ppm sized buckets) in the range of 0.50–10 ppm. The area within each bucket was normalized to the total intensity. The areas of the buckets in the regions 4.50–5.20 and 3.28–3.40 ppm, corresponding to the residual signals of water and methanol, respectively, were excluded. The matrices with dimensions of *samples* × *number of buckets* containing the normalized spectral intensities were used as input for the chemometric analysis.

### 3.5. Chemometric Methods

An exploratory analysis of the experimental data obtained from ^1^H NMR and EEM fluorescence (unfolded matrices) was performed by means of Principal Component Analysis. For the geographical origin characterization, the data were analyzed by means of a Soft Independent Modeling of Class Analogy (SIMCA) chemometric model. SIMCA was originally developed by Svante Wold in 1976 [81,82], and it has been extensively used as a supervised pattern recognition method in combination with different experimental techniques for geographical origin authentication [83,84,85], quality assessment [86,87], and fraud detection [88,89,90]. SIMCA consists of building a PCA model to describe the variance within each class separately. Each class was described by its own PCA model, and the boundaries were defined by confidence limits based on the residual variance. The experimental data were projected onto these models to determine the class membership, allowing for the identification and characterization of distinct groups within the dataset. The classification rule is defined by the distance of the sample from the class model, which is calculated from the normalized Q residuals and normalized Hotelling T^2^ values. Q residuals and Hotelling T^2^ are normalized over their 95% confidence limits. The performance of the SIMCA model was evaluated by calculating the accuracy (ratio of correctly assigned samples), sensitivity, and specificity of the classification, which are defined as follows:

$$Sensitivity=\frac{TP}{TP+FN}$$

$$Specificity=\frac{TN}{TN+FP}$$

where *TPs* are the true positives, *FNs* the false negatives, *TNs* the true negatives, and *FPs* the false positives. Sensitivity measures how well target class samples are correctly recognized, while specificity represents how many non-target class samples are rejected by the model built for the investigated class [91].

The distance threshold is not fixed, since its value is tuned and optimized in order to maximize class specificity and sensitivity, following the approach of Vitale et al. [92].

The number of principal components to be retained in order to build the PCA model for each class was selected on the basis of the minimum of root mean square error in cross-validation (RMSECV, Leave-one-out cross-validation) and the maximum of sensitivity estimated in cross-validation [93]. The model performances were evaluated using a test set validation: each dataset was split into a calibration and a validation set using the duplex algorithm with a splitting ratio of 80:20 [94].

All the measurements (both ^1^H NMR and fluorescence) were conducted in triplicate to ensure analytical reproducibility. Moreover, a careful preliminary inspection of the datasets was performed to detect and remove outliers prior to modeling, based on leverage and Q-residual diagnostics following standard PCA-based approaches. As for preprocessing, both ^1^H NMR and EEM datasets were mean-centered prior to the chemometric analysis.

Parallel Factor Analysis (PARAFAC) decomposition was applied to decompose the three-dimensional data into individual fluorescent components based on their spectral signatures. The algorithm works by fitting the data into a trilinear model, assuming that the fluorescence intensity is the product of excitation and emission spectra for each component, along with their relative concentrations [37,95]. The decomposition generates three sets of bidimensional matrices, or loadings: sample loadings (also referred to as scores), excitation loadings, and emission loadings. The sample loadings correspond to the relative concentration of each component across the different samples, providing insights into how the components vary across the dataset, while excitation and emission loadings represent the fluorescence excitation and emission spectra of each component, respectively. The optimal number of components was determined on the basis of different parameters, namely the central consistency diagnostic criterion (CORe CONsistency DIAgnostic, CORCONDIA), the percentage of variance explained by the model, and visual inspection of the recovered spectral and residual profiles. Non-negative constraints were applied for all the modes.

Although the PARAFAC approach might be more suitable for identifying which fluorophores are present in the samples, contributing at the same time to geographical authentication, using unfolded data appeared more effective for the straightforward classification of olive oil, fruit, and leaf samples [96]. This difference can be attributed to the fact that the unfolded matrix retains all available data, whereas PARAFAC reduces the amount of information and is highly influenced by the number of components chosen, which may lower classification accuracy [97]. However, the major drawback of multidimensional unfolding methods is the higher complexity and the more difficult interpretation of the generated outputs, which must be refolded to restore the original modes.

All the calculations were performed in a MATLAB environment (MATLAB R2023b version, The MathWorks Inc., Natick, MA, USA) using PCA [98] and classification toolboxes [99] for MATLAB from Milano Chemometrics and QSAR Research Group (https://michem.unimib.it/, accessed on 3 February 2025).

### 3.6. Data Fusion

The multi-block analysis on the data from the two different spectroscopic techniques (^1^H NMR spectra and unfolded fluorescence matrices) was performed according to a mid-level data fusion based on the ComDim (Common Dimension) method, a particular application of the Common Components and Specific Weights Analysis (CCSWA) procedure developed by Qannari [48,100]. The first step is the organization of the data into two different blocks, corresponding to the individual analytical techniques. The ^1^H NMR data and unfolded EEMs are normalized by dividing each point by the square root of the sum of squared values [101]. After concatenation, each block is normalized by its Frobenius norm so that they all have the same total variance. The ComDim approach focuses on the variance-covariance matrices of the samples, which are all of the same dimensions. This allows for the calculation of a weighted sum of these matrices, from which the first normalized principal component, referred to as the “Common Dimension” (CD), is extracted. The algorithm then iteratively adjusts the weight, or “salience,” of each data block for the identified CD. After the first CD is computed, each data block matrix is deflated, and the process is repeated to calculate subsequent CDs. As a result, each CD represents the first principal component of the weighted sum of the variance-covariance matrices of the deflated blocks [48,102,103,104]. The resulting scores (i.e., the extracted common components) and loadings provide a direct visualization of sample distribution and variable contribution, enhancing the understanding of sample similarities, clustering, and correlations with the geographical origin.

To support the classification of leaf and drupe samples according to their geographical origin, a SIMCA-like model was developed based on the results of the ComDim multiblock analysis. In this approach, global scores and residuals derived from the ComDim model—applied to the integrated dataset combining ^1^H NMR and EEM fluorescence spectroscopy—are used to calculate two metrics, the score distance (SD) and the orthogonal distance (OD), respectively, for each training sample of the target class. The ComDim scores were used as input without pre-processing. These metrics were combined into a single reduced distance, used to quantify the degree of class membership for each sample. The threshold value is optimized in order to maximize class specificity and sensitivity [92]. New samples were projected onto the ComDim model, and their reduced distance values were compared to the threshold to determine class inclusion. This methodology is analogous to the multiblock extension of the one-class classifiers that integrates ComDim with the data-driven SIMCA model originally proposed by Galván and co-workers [105]. Model performance was evaluated in terms of accuracy, sensitivity, and specificity, based on a validation strategy involving the division of the dataset into separate calibration and external test sets, as in the case of single block-based SIMCA. All the calculations were performed using the MBA-GUI toolbox for MATLAB [106] and in-house MATLAB scripts.

## 4. Conclusions

This study highlights the potential of ^1^H NMR and EEM fluorescence spectroscopy in combination with single- and multi-block chemometric methods for the geographical authentication of olive leaves and drupes. Individually, each technique provided robust classification results, with ^1^H NMR demonstrating its strength in identifying key metabolites that provide the major contribution to geographic origin differentiation, such as secoiridoids and organic acids. This is particularly evident in the case of olive leaves, where the SIMCA modeling provides excellent classification performances for the investigated geographical area. Similarly, through EEM fluorescence spectroscopy, the fluorescence profiles of the different matrices can be effectively characterized by identifying classes of emitting compounds like tocopherols, polyphenols, and chlorogenic acid, which can be used as distinct markers for sample classification.

A key novelty of this work is the application of EEM fluorescence spectroscopy for the geographical origin authentication of olive drupes and leaves, an approach that, to the best of our knowledge, has not been previously reported. The three-dimensional spectral data can be refolded and effectively used as inputs for chemometric models to discriminate between subregional areas, providing a powerful alternative for food and vegetable matrices authentication. This novel application of a relatively simple and low-cost technique, such as EEM fluorescence (especially, if compared to NMR), expands the analytical possibilities for olive product classification, offering a complementary tool alongside NMR-based metabolic profiling.

This is particularly evident, also looking at the most relevant result of the present study, i.e., the integration of these techniques through data fusion further enhanced the sample differentiation by leveraging the complementary strengths of each method. Multivariate statistical analysis based on multi-technique datasets is still relatively unexploited in the field of geographical assessment of fruit and plant matrices related to agrifood products. Here, the data fusion approach, which was based on the ComDim algorithm, allowed for an enhanced sample visualization at an exploratory level, and improved the robustness of classification models when combined with SIMCA into a multiblock one-class modeling strategy, especially for the drupe samples. In this case, in fact, the SIMCA model computed on individual datasets presented some limitations, which were significantly overcome with the inclusion of both spectroscopic data, using the aforementioned multi-block approach. This result emphasizes the importance of evaluating data fusion not as a universal improvement, but as a matrix- and context-dependent strategy with the potential to enhance model robustness when single-block approaches fall short.

Overall, this comprehensive approach underscores the value of combining advanced spectroscopic techniques with chemometric tools for food authentication.

Nonetheless, it should be acknowledged that the present study is based on an exploratory sampling design involving three subregions within Tuscany, Italy. While this provides a solid and controlled framework for assessing classification performance at the subregional level, the scope is primarily focused on feasibility rather than broad generalizability. The classification models developed herein have demonstrated their effectiveness within this localized context; however, their extension to wider geographical areas or different countries will require further validation. This is particularly relevant given that the chemical profiles of olive matrices are known to be influenced by several environmental and agronomic factors, including harvest time, climate conditions, soil characteristics, and cultivation practices.

Future studies will aim to extend the analysis to the final products, i.e., olive oils, and further refine these methodologies to address current limitations and strengthen the models’ predictive ability.

## Figures and Tables

**Table 1:** SIMCA results obtained for the ^1^H NMR and the EEM fluorescence data of the olive leaf and drupe extracts for the Chianti–Siena area.

SIMCA Model for Chianti–Siena Region							
	Training			Test			
	Accuracy (%)	Sensitivity (%)	Specificity (%)	Accuracy (%)	Sensitivity (%)	Specificity (%)	Explained Variance (%)
Leaves ^1^H NMR	100	100	100	83	100	50	97
Leaves EEM	84	100	43	83	100	50	94
Drupes ^1^H NMR	88	90	82	70	75	50	95
Drupes EEM	76	77	73	50	50	50	90

**Table 2:** Fluorescent properties of compounds detected in olive leaves and drupes.

Analyzed Matrix	Fluorescence Region	Compound	Excitation Wavelength (nm)	Emission Wavelength (nm)
Leaves	Region A	Chlorophyll a	430, 670	675
		Pheophytin a	410, 660	670
		Chlorophyll b	460, 650	665
	Region B	Chlorogenic acid	320	435
		Phenolic compounds	280	<370
		Tocopherols	360	465
Drupes	Full map	Catechin/epicatechin	280	315
		Tocopherols	340	450
		Phenolic compounds	230	310

**Table 3:** Classification performances obtained with the combined ComDim-based SIMCA approach for the ^1^H NMR and the EEM fluorescence data of the olive leaf and drupe extracts for the Chianti–Siena area.

ComDim-Based SIMCA Multiblock Model for Chianti–Siena Region							
	Training			Test			
	Accuracy (%)	Sensitivity (%)	Specificity (%)	Accuracy (%)	Sensitivity (%)	Specificity (%)	Explained Variance (%)
Leaves	84	83	86	83	100	50	95
Drupes	86	90	73	90	100	50	93

## Data Availability

Data is contained within the article or Supplementary Material.

## Supplementary Materials

The following supporting information can be downloaded at: https://www.mdpi.com/article/10.3390/molecules30153208/s1, Table S1. Description of the analyzed drupe and leaf samples, including climatic and environmental data; Table S2. ^1^H NMR assignment for the olive leaf extracts; Figure S1. PCA loading plot for ^1^H NMR data of the olive leaf samples (a) and 2D score plots: PC1 vs. PC2 (b), PC1 vs. PC3 (c), and PC2 vs. PC3 (d); Figure S2. SIMCA normalized distances from ^1^H NMR data of the olive leaf samples for the modeled Chianti–Siena region; Figure S3. Sample, excitation, and emission PARAFAC loadings for the olive leaf samples in spectral Region A; Figure S4. Sample, excitation, and emission PARAFAC loadings for the olive leaf samples in spectral Region B; Figure S5. Three-dimensional PCA score plot for EEM (Region B) data of the olive leaf samples; Figure S6. PCA loading plot for EEM (Region B) data of the olive leaf samples (a) and 2D score plots: PC1 vs. PC2 (b), PC1 vs. PC3 (c), and PC2 vs. PC3 (d); Figure S7. SIMCA normalized distances from EEM data of the olive leaf samples for the modeled Chianti–Siena region. The right panel shows a magnified view of the distance distribution; Table S3. ^1^H NMR assignment for the drupe extracts; Figure S8. Three-dimensional PCA score plot for ^1^H NMR data of the olive drupe samples; Figure S9. PCA loading plot for ^1^H NMR data of the olive drupe samples (a) and 2D score plots: PC1 vs. PC2 (b), PC1 vs. PC3 (c), and PC2 vs. PC3 (d); Figure S10. SIMCA normalized distances from EEM data of the olive drupe samples for the modeled Chianti–Siena region; Figure S11. Sample, excitation, and emission PARAFAC loadings for the drupe samples; Figure S12. Three-dimensional PCA score plot for EEM data of the olive drupe samples; Figure S13. PCA loading plot for EEM data of the olive drupe samples (a) and 2D score plots: PC1 vs. PC2 (b), PC1 vs. PC3 (c), and PC2 vs. PC3 (d); Figure S14. SIMCA normalized distances from EEM data of the olive drupe samples for the modeled Chianti–Siena region; Figure S15. ComDim salience plot for the leaf samples (a) and 2D ComDim score plots: CD1 vs. CD2 (b), CD1 vs. CD3 (c), and CD2 vs. CD3 (d); Figure S16. ComDim loadings for the ^1^H NMR (left panel) and EEM block (right panel) for each Common Dimension calculated for the olive leaves dataset; Figure S17. ComDim salience plot for the drupe samples (a) and 2D ComDim score plots: CD1 vs. CD2 (b), CD1 vs. CD3 (c), and CD2 vs. CD3 (d); Figure S18. ComDim loadings for the ^1^H NMR (left panel) and EEM block (right panel) for each Common Dimension calculated for the olive drupe dataset; Figure S19. Reduced distances calculated from the ComDim-based SIMCA multiblock model for the leaf (a) and drupe (b) samples for the Chianti–Siena region. References [20,51,52,54,55,56,58,59,60,72,73,74,75,76] are cited in the Supplementary Materials.

## Abbreviations

The following abbreviations are used in this manuscript:

^1^H NMR	Proton Nuclear Magnetic Resonance
CCSWA	Common Components and Specific Weights Analysis
CD	Common Dimension
ComDim	Common Dimensions algorithm
CORCONDIA	Core Consistency Diagnostic
DA	Discriminant Analysis
EEM	Excitation–Emission Matrix
FID	Fourier Transformation of the Free Induction Decay
FN	False Negative
FP	False Positive
HPLC-HRMS	High-Performance Liquid Chromatography-High Resolution Mass Spectrometry
LDA	Linear Discriminant Analysis
OD	Orthogonal Distance
PARAFAC	Parallel Factor Analysis
PCA	Principal Component Analysis
PDO	Protected Designation Of Origin
RMSECV	Minimum of Root Mean Squared Error in Cross-Validation
SD	Score Distance
SIMCA	Soft Independent Modeling of Class Analogy
TP	True Positive
TSP-D4	3-(trimethylsilyl)propionic-2,2,3,3-d_4_ acid sodium salt
UV-Vis	UV–Visible

## References

[1] https://www.internationaloliveoil.org/wp-content/uploads/2023/12/IOC-Olive-Oil-Dashboard.html.

[2] Gagour J., Hallouch O., Asbbane A., Bijla L., Laknifli A., Lee L., Zengin G., Bouyahya A., Sakar E.H., Gharby S. (2024). A Review of Recent Progresses on Olive Oil Chemical Profiling, Extraction Technology, Shelf-life, and Quality Control. Chem. Biodivers..

[3] Lazzerini C., Domenici V. (2017). Pigments in Extra-Virgin Olive Oils Produced in Tuscany (Italy) in Different Years. Foods.

[4] Blasi F., Ianni F., Cossignani L. (2024). Phenolic Profiling for Geographical and Varietal Authentication of Extra Virgin Olive Oil. Trends Food Sci. Technol..

[5] Lozano-Castellón J., López-Yerena A., Domínguez-López I., Siscart-Serra A., Fraga N., Sámano S., López-Sabater C., Lamuela-Raventós R.M., Vallverdú-Queralt A., Pérez M. (2022). Extra Virgin Olive Oil: A Comprehensive Review of Efforts to Ensure Its Authenticity, Traceability, and Safety. Compr. Rev. Food Sci. Food Saf..

[6] Nardin R., Tamasi G., Baglioni M., Fattori G., Boldrini A., Esposito R., Rossi C. (2024). Combining Metal(Loid) and Secondary Metabolite Levels in *Olea europaea* L. Samples for Geographical Identification. Foods.

[7] Di Vaio C., Nocerino S., Paduano A., Sacchi R. (2013). Influence of Some Environmental Factors on Drupe Maturation and Olive Oil Composition. J. Sci. Food Agric..

[8] Morelló J.-R., Romero M.-P., Motilva M.-J. (2004). Effect of the Maturation Process of the Olive Fruit on the Phenolic Fraction of Drupes and Oils from Arbequina, Farga, and Morrut Cultivars. J. Agric. Food Chem..

[9] Borghini F., Tamasi G., Loiselle S.A., Baglioni M., Ferrari S., Bisozzi F., Costantini S., Tozzi C., Riccaboni A., Rossi C. (2024). Phenolic Profiles in Olive Leaves from Different Cultivars in Tuscany and Their Use as a Marker of Varietal and Geographical Origin on a Small Scale. Molecules.

[10] Nardin R., Tamasi G., Baglioni M., Bisozzi F., Consumi M., Costa J., Fattori G., Tozzi C., Riccaboni A., Rossi C. (2024). Determination of Elemental Content in Vineyard Soil, Leaves, and Grapes of Sangiovese Grapes from the Chianti Region Using ICP-MS for Geographical Identification. ACS Food Sci. Technol..

[11] Pucci E., Palumbo D., Puiu A., Lai A., Fiorani L., Zoani C. (2022). Characterization and Discrimination of Italian Olive (*Olea europaea* Sativa) Cultivars by Production Area Using Different Analytical Methods Combined with Chemometric Analysis. Foods.

[12] Meenu M., Cai Q., Xu B. (2019). A Critical Review on Analytical Techniques to Detect Adulteration of Extra Virgin Olive Oil. Trends Food Sci. Technol..

[13] Gómez-Caravaca A.M., Maggio R.M., Cerretani L. (2016). Chemometric Applications to Assess Quality and Critical Parameters of Virgin and Extra-Virgin Olive Oil. A Review. Anal. Chim. Acta.

[14] Varzakas T., Tarapoulouzi M., Agriopoulou S. (2022). Chemometrics and Authenticity of Foods of Plant Origin.

[15] Cappelli A., Cividino S., Redaelli V., Tripodi G., Aiello G., Velotto S., Zaninelli M. (2024). Applying Spectroscopies, Imaging Analyses, and Other Non-Destructive Techniques to Olives and Extra Virgin Olive Oil: A Systematic Review of Current Knowledge and Future Applications. Agriculture.

[16] Kalogiouri N.P., Aalizadeh R., Dasenaki M.E., Thomaidis N.S. (2020). Authentication of Greek PDO Kalamata Table Olives: A Novel Non-Target High Resolution Mass Spectrometric Approach. Molecules.

[17] Tamasi G., Baratto M.C., Bonechi C., Byelyakova A., Pardini A., Donati A., Leone G., Consumi M., Lamponi S., Magnani A. (2019). Chemical Characterization and Antioxidant Properties of Products and By-Products from *Olea europaea* L.. Food Sci. Nutr..

[18] Cangeloni L., Bonechi C., Leone G., Consumi M., Andreassi M., Magnani A., Rossi C., Tamasi G. (2022). Characterization of Extracts of Coffee Leaves (*Coffea arabica* L.) by Spectroscopic and Chromatographic/Spectrometric Techniques. Foods.

[19] Girelli C.R., Del Coco L., Zelasco S., Salimonti A., Conforti F.L., Biagianti A., Barbini D., Fanizzi F.P. (2018). Traceability of “Tuscan PGI” Extra Virgin Olive Oils by 1H NMR Metabolic Profiles Collection and Analysis. Metabolites.

[20] Beteinakis S., Papachristodoulou A., Gogou G., Katsikis S., Mikros E., Halabalaki M. (2020). NMR-Based Metabolic Profiling of Edible Olives—Determination of Quality Parameters. Molecules.

[21] Dais P., Hatzakis E. (2015). Analysis of Bioactive Microconstituents in Olives, Olive Oil and Olive Leaves by NMR Spectroscopy: An Overview of the Last Decade. Olive and Olive Oil Bioactive Constituents.

[22] Mannina L., Sobolev A.P. (2011). High Resolution NMR Characterization of Olive Oils in Terms of Quality, Authenticity and Geographical Origin. Magn. Reson. Chem..

[23] Beteinakis S., Papachristodoulou A., Kolb P., Rösch P., Schwarzinger S., Mikros E., Halabalaki M. (2023). NMR-Based Metabolite Profiling and the Application of STOCSY toward the Quality and Authentication Assessment of European EVOOs. Molecules.

[24] Calò F., Girelli C.R., Wang S.C., Fanizzi F.P. (2022). Geographical Origin Assessment of Extra Virgin Olive Oil via NMR and MS Combined with Chemometrics as Analytical Approaches. Foods.

[25] Maestrello V., Solovyev P., Bontempo L., Mannina L., Camin F. (2022). Nuclear Magnetic Resonance Spectroscopy in Extra Virgin Olive Oil Authentication. Compr. Rev. Food Sci. Food Saf..

[26] Dais P., Hatzakis E. (2013). Quality Assessment and Authentication of Virgin Olive Oil by NMR Spectroscopy: A Critical Review. Anal. Chim. Acta.

[27] Tamasi G., Bonechi C., Leone G., Andreassi M., Consumi M., Sangiorgio P., Verardi A., Rossi C., Magnani A. (2021). Varietal and Geographical Origin Characterization of Peaches and Nectarines by Combining Analytical Techniques and Statistical Approach. Molecules.

[28] Sikorska E., Khmelinskii I., Sikorski M. (2019). Fluorescence Spectroscopy and Imaging Instruments for Food Quality Evaluation. Evaluation Technologies for Food Quality.

[29] Ranaweera R.K.R., Gilmore A.M., Capone D.L., Bastian S.E.P., Jeffery D.W. (2021). Authentication of the Geographical Origin of Australian Cabernet Sauvignon Wines Using Spectrofluorometric and Multi-Element Analyses with Multivariate Statistical Modelling. Food Chem..

[30] Sádecká J., Uríčková V., Jakubíková M., Stauffer M.T. (2016). Fluorescence Spectroscopy for the Analysis of Spirit Drinks. Applications of Molecular Spectroscopy to Current Research in the Chemical and Biological Sciences.

[31] Sikorska E., Gliszczyńska-Świgło A., Khmelinskii I., Sikorski M. (2005). Synchronous Fluorescence Spectroscopy of Edible Vegetable Oils. Quantification of Tocopherols. J. Agric. Food Chem..

[32] Latchoumane L., Alary K., Minier J., Davrieux F., Lugan R., Chillet M., Roger J.-M. (2022). Front-Face Fluorescence Spectroscopy and Feature Selection for Fruit Classification Based on N-CovSel Method. Front. Anal. Sci..

[33] Lenhardt L., Zeković I., Dramićanin T., Milićević B., Burojević J., Dramićanin M.D. (2017). Characterization of Cereal Flours by Fluorescence Spectroscopy Coupled with PARAFAC. Food Chem..

[34] Kulmyrzaev A.A., Levieux D., Dufour É. (2005). Front-Face Fluorescence Spectroscopy Allows the Characterization of Mild Heat Treatments Applied to Milk. Relations with the Denaturation of Milk Proteins. J. Agric. Food Chem..

[35] Abbas K., Karoui R., Aït-Kaddour A. (2012). Application of Synchronous Fluorescence Spectroscopy for the Determination of Some Chemical Parameters in PDO French Blue Cheeses. Eur. Food Res. Technol..

[36] Sikorska E., Khmelinskii I., Sikorski M., Boskou D. (2012). Analysis of Olive Oils by Fluorescence Spectroscopy: Methods and Applications. Olive Oil—Constituents, Quality, Health Properties and Bioconversions.

[37] Murphy K.R., Stedmon C.A., Graeber D., Bro R. (2013). Fluorescence Spectroscopy and Multi-Way Techniques. PARAFAC. Anal. Methods.

[38] Amigo J.M., Marini F. (2013). Multiway Methods. Data Handling in Science and Technology.

[39] Morais C.L.M., Lima K.M.G. (2017). Comparing Unfolded and Two-Dimensional Discriminant Analysis and Support Vector Machines for Classification of EEM Data. Chemom. Intell. Lab. Syst..

[40] Al Riza D.F., Kondo N., Rotich V.K., Perone C., Giametta F. (2021). Cultivar and Geographical Origin Authentication of Italian Extra Virgin Olive Oil Using Front-Face Fluorescence Spectroscopy and Chemometrics. Food Control.

[41] De Paulo E.H., Magalhães G.B., Moreira M.P.B., Nascimento M.H.C., Heringer O.A., Filgueiras P.R., Ferrão M.F. (2024). Classification of Water by Bacterial Presence Using Chemometrics Associated with Excitation-Emission Matrix Fluorescence Spectroscopy. Microchem. J..

[42] Suciu R.-C., Zarbo L., Guyon F., Magdas D.A. (2019). Application of Fluorescence Spectroscopy Using Classical Right Angle Technique in White Wines Classification. Sci. Rep..

[43] Katerinopoulou K., Kontogeorgos A., Salmas C.E., Patakas A., Ladavos A. (2020). Geographical Origin Authentication of Agri-Food Products: A Review. Foods.

[44] Cozzolino D. (2012). Recent Trends on the Use of Infrared Spectroscopy to Trace and Authenticate Natural and Agricultural Food Products. Appl. Spectrosc. Rev..

[45] Garrido-Cuevas M.-M., Garrido-Varo A.-M., Marini F., Sánchez M.-T., Pérez-Marín D. (2025). Enhancing Virgin Olive Oil Authentication with Bayesian Probabilistic Models and near Infrared Spectroscopy. J. Food Eng..

[46] Biancolillo A., Boqué R., Cocchi M., Marini F. (2019). Data Fusion Strategies in Food Analysis. Data Handling in Science and Technology.

[47] El Ghaziri A., Cariou V., Rutledge D.N., Qannari E.M. (2016). Analysis of Multiblock Datasets Using ComDim: Overview and Extension to the Analysis of (K + 1) Datasets. J. Chemom..

[48] Rosa L.N., De Figueiredo L.C., Bonafé E.G., Coqueiro A., Visentainer J.V., Março P.H., Rutledge D.N., Valderrama P. (2017). Multi-Block Data Analysis Using ComDim for the Evaluation of Complex Samples: Characterization of Edible Oils. Anal. Chim. Acta.

[49] Costantini E.A.C., Barbetti R., Bucelli P., L’Abate G., Lelli L., Pellegrini S., Storchi P. (2006). Land Peculiarities of the Vine Cultivation Areas in the Province of Siena (Italy), with Reference to the Viticultural and Oenological Results of Sangiovese Vine. Boll. Della Soc. Geol. Ital. Suppl..

[50] Rahmanian N., Jafari S.M., Wani T.A. (2015). Bioactive Profile, Dehydration, Extraction and Application of the Bioactive Components of Olive Leaves. Trends Food Sci. Technol..

[51] Girelli C.R., Angilè F., Del Coco L., Migoni D., Zampella L., Marcelletti S., Cristella N., Marangi P., Scortichini M., Fanizzi F.P. (2019). 1H-NMR Metabolite Fingerprinting Analysis Reveals a Disease Biomarker and a Field Treatment Response in Xylella Fastidiosa Subsp. Pauca-Infected Olive Trees. Plants.

[52] Girelli C.R., Hussain M., Verweire D., Oehl M.C., Massana-Codina J., Avendaño M.S., Migoni D., Scortichini M., Fanizzi F.P. (2022). Agro-Active Endo-Therapy Treated Xylella Fastidiosa Subsp. Pauca-Infected Olive Trees Assessed by the First 1H-NMR-Based Metabolomic Study. Sci. Rep..

[53] Benavente-García O., Castillo J., Lorente J., Ortuño A., Del Rio J.A. (2000). Antioxidant Activity of Phenolics Extracted from *Olea europaea* L. Leaves. Food Chem..

[54] Huertas-Alonso A.J., Gavahian M., González-Serrano D.J., Hadidi M., Salgado-Ramos M., Sánchez-Verdú M.P., Simirgiotis M.J., Barba F.J., Franco D., Lorenzo J.M. (2021). Valorization of Wastewater from Table Olives: NMR Identification of Antioxidant Phenolic Fraction and Microwave Single-Phase Reaction of Sugary Fraction. Antioxidants.

[55] Goulas V., Exarchou V., Troganis A.N., Psomiadou E., Fotsis T., Briasoulis E., Gerothanassis I.P. (2009). Phytochemicals in Olive-leaf Extracts and Their Antiproliferative Activity against Cancer and Endothelial Cells. Mol. Nutr. Food Res..

[56] Karkoula E., Skantzari A., Melliou E., Magiatis P. (2012). Direct Measurement of Oleocanthal and Oleacein Levels in Olive Oil by Quantitative^1^ H NMR. Establishment of a New Index for the Characterization of Extra Virgin Olive Oils. J. Agric. Food Chem..

[57] Filardo S., Roberto M., Di Risola D., Mosca L., Di Pietro M., Sessa R. (2024). *Olea europaea* L-Derived Secoiridoids: Beneficial Health Effects and Potential Therapeutic Approaches. Pharmacol. Ther..

[58] Kalampaliki A.D., Giannouli V., Skaltsounis A.-L., Kostakis I.K. (2019). A Three-Step, Gram-Scale Synthesis of Hydroxytyrosol, Hydroxytyrosol Acetate, and 3,4-Dihydroxyphenylglycol. Molecules.

[59] Wishart D.S., Knox C., Guo A.C., Eisner R., Young N., Gautam B., Hau D.D., Psychogios N., Dong E., Bouatra S. (2009). HMDB: A Knowledgebase for the Human Metabolome. Nucleic Acids Res..

[60] Agatonovic-Kustrin S., Gegechkori V., Morton D.W., Tucci J., Mohammed E.U.R., Ku H. (2022). The Bioprofiling of Antibacterials in Olive Leaf Extracts via Thin Layer Chromatography-Effect Directed Analysis (TLC-EDA). J. Pharm. Biomed. Anal..

[61] Guimet F., Ferré J., Boqué R., Vidal M., Garcia J. (2005). Excitation–Emission Fluorescence Spectroscopy Combined with Three-Way Methods of Analysis as a Complementary Technique for Olive Oil Characterization. J. Agric. Food Chem..

[62] Lia F., Formosa J.P., Zammit-Mangion M., Farrugia C. (2020). The First Identification of the Uniqueness and Authentication of Maltese Extra Virgin Olive Oil Using 3D-Fluorescence Spectroscopy Coupled with Multi-Way Data Analysis. Foods.

[63] Guimet F., Boqué R., Ferré J. (2004). Cluster Analysis Applied to the Exploratory Analysis of Commercial Spanish Olive Oils by Means of Excitation−Emission Fluorescence Spectroscopy. J. Agric. Food Chem..

[64] Martín-Tornero E., Durán Martín-Merás I., Espinosa Mansilla A., Almeida Lopes J., Nuno Mendes De Jorge Páscoa R. (2022). Geographical Discrimination of Grapevine Leaves Using Fibre Optic Fluorescence Data and Chemometrics. Determination of Total Polyphenols and Chlorophylls along Different Vegetative Stages. Microchem. J..

[65] Guimet F., Ferré J., Boqué R., Rius F.X. (2004). Application of Unfold Principal Component Analysis and Parallel Factor Analysis to the Exploratory Analysis of Olive Oils by Means of Excitation–Emission Matrix Fluorescence Spectroscopy. Anal. Chim. Acta.

[66] Giuliani A., Cerretani L., Cichelli A. (2011). Chlorophylls in Olive and in Olive Oil: Chemistry and Occurrences. Crit. Rev. Food Sci. Nutr..

[67] Galeano Díaz T., Durán Merás I., Correa C.A., Roldán B., Rodríguez Cáceres M.I. (2003). Simultaneous Fluorometric Determination of Chlorophylls a and b and Pheophytins a and b in Olive Oil by Partial Least-Squares Calibration. J. Agric. Food Chem..

[68] Navarro-Orcajada S., Matencio A., Vicente-Herrero C., García-Carmona F., López-Nicolás J.M. (2021). Study of the Fluorescence and Interaction between Cyclodextrins and Neochlorogenic Acid, in Comparison with Chlorogenic Acid. Sci. Rep..

[69] Hernández-Sánchez N., Lleó L., Diezma B., Correa E.C., Sastre B., Roger J.-M. (2021). Multiblock Analysis Applied to Fluorescence and Absorbance Spectra to Estimate Total Polyphenol Content in Extra Virgin Olive Oil. Foods.

[70] Zandomeneghi M., Carbonaro L., Caffarata C. (2005). Fluorescence of Vegetable Oils: Olive Oils. J. Agric. Food Chem..

[71] Quintanilla-Casas B., Rinnan Å., Romero A., Guardiola F., Tres A., Vichi S., Bro R. (2022). Using Fluorescence Excitation-Emission Matrices to Predict Bitterness and Pungency of Virgin Olive Oil: A Feasibility Study. Food Chem..

[72] Tasnuva S.T., Qamar U.A., Ghafoor K., Sahena F., Jahurul M.H.A., Rukshana A.H., Juliana M.J., Al-Juhaimi F.Y., Jalifah L., Jalal K.C.A. (2019). α-Glucosidase Inhibitors Isolated from *Mimosa pudica* L.. Nat. Prod. Res..

[73] Wang L., Li X., Zhang S., Lu W., Liao S., Liu X., Shan L., Shen X., Jiang H., Zhang W. (2012). Natural Products as a Gold Mine for Selective Matrix Metalloproteinases Inhibitors. Bioorg. Med. Chem..

[74] Esposito A., De Luca P.F., Graziani V., D’Abrosca B., Fiorentino A., Scognamiglio M. (2021). Phytochemical Characterization of Olea Europaea L. Cultivars of Cilento National Park (South Italy) through NMR-Based Metabolomics. Molecules.

[75] De Cássia Lemos Lima R., Kongstad K.T., Kato L., José das Silva M., Franzyk H., Staerk D. (2018). High-Resolution PTP1B Inhibition Profiling Combined with HPLC-HRMS-SPE-NMR for Identification of PTP1B Inhibitors from Miconia Albicans. Molecules.

[76] Beteinakis S., Papachristodoulou A., Stathopoulos P., Mikros E., Halabalaki M. (2024). A Multilevel LC-HRMS and NMR Correlation Workflow towards Foodomics Advancement: Application in Table Olives. Talanta.

[77] Du C., Ma C., Gu J., Li L., Zhu C., Chen L., Wang T., Chen G. (2020). Rapid Determination of Catechin Content in Black Tea by Fluorescence Spectroscopy. J. Spectrosc..

[78] Gonçalves T.R., Teixeira G.G., Santos P.M., Matsushita M., Valderrama P. (2023). Excitation-Emission Matrices and PARAFAC in the Investigation of the Bioactive Compound Effects from the Flavoring Process in Olive Oils. Microchem. J..

[79] Andersson C.A., Bro R. (2000). The N-Way Toolbox for MATLAB. Chemom. Intell. Lab. Syst..

[80] Jacob D., Deborde C., Lefebvre M., Maucourt M., Moing A. (2017). NMRProcFlow: A Graphical and Interactive Tool Dedicated to 1D Spectra Processing for NMR-Based Metabolomics. Metabolomics.

[81] Wold S. (1976). Pattern Recognition by Means of Disjoint Principal Components Models. Pattern Recognit..

[82] Wold S., Sjöström M. (1977). SIMCA: A Method for Analyzing Chemical Data in Terms of Similarity and Analogy. Chemometrics: Theory and Application.

[83] Tahir H.E., Arslan M., Komla Mahunu G., Adam Mariod A., Hashim S.B.H., Xiaobo Z., Jiyong S., El-Seedi H.R., Musa T.H. (2022). The Use of Analytical Techniques Coupled with Chemometrics for Tracing the Geographical Origin of Oils: A Systematic Review (2013–2020). Food Chem..

[84] Marini F., Bucci R., Magrì A.L., Magrì A.D. (2010). An Overview of the Chemometric Methods for the Authentication of the Geographical and Varietal Origin of Olive Oils. Olives and Olive Oil in Health and Disease Prevention.

[85] Masetti O., Sorbo A., Nisini L. (2021). NMR Tracing of Food Geographical Origin: The Impact of Seasonality, Cultivar and Production Year on Data Analysis. Separations.

[86] Marini F., Biancolillo A. (2023). Application of Spectroscopy in Food Analysis: Volume II. Appl. Sci..

[87] Zaldarriaga Heredia J., Wagner M., Jofré F.C., Savio M., Azcarate S.M., Camiña J.M. (2023). An Overview on Multi-Elemental Profile Integrated with Chemometrics for Food Quality Assessment: Toward New Challenges. Crit. Rev. Food Sci. Nutr..

[88] De Angelis D., Summo C., Pasqualone A., Faccia M., Squeo G. (2024). Advancements in Food Authentication Using Soft Independent Modelling of Class Analogy (SIMCA): A Review. Food Qual. Saf..

[89] Biancolillo A., Marini F., Ruckebusch C., Vitale R. (2020). Chemometric Strategies for Spectroscopy-Based Food Authentication. Appl. Sci..

[90] Rodionova O.Y., Pomerantsev A.L. (2020). Chemometric Tools for Food Fraud Detection: The Role of Target Class in Non-Targeted Analysis. Food Chem..

[91] Vitale R., Cocchi M., Biancolillo A., Ruckebusch C., Marini F. (2023). Class Modelling by Soft Independent Modelling of Class Analogy: Why, When, How? A Tutorial. Anal. Chim. Acta.

[92] Vitale R., Marini F., Ruckebusch C. (2018). SIMCA Modeling for Overlapping Classes: Fixed or Optimized Decision Threshold?. Anal. Chem..

[93] Li Vigni M., Durante C., Michelini S., Nocetti M., Cocchi M. (2020). Preliminary Assessment of Parmigiano Reggiano Authenticity by Handheld Raman Spectroscopy. Foods.

[94] Snee R.D. (1977). Validation of Regression Models: Methods and Examples. Technometrics.

[95] Bro R. (1997). PARAFAC. Tutorial and Applications. Chemom. Intell. Lab. Syst..

[96] Durán Merás I., Domínguez Manzano J., Airado Rodríguez D., Muñoz De La Peña A. (2018). Detection and Quantification of Extra Virgin Olive Oil Adulteration by Means of Autofluorescence Excitation-Emission Profiles Combined with Multi-Way Classification. Talanta.

[97] Ríos-Reina R., Salatti-Dorado J.Á., Ortiz-Romero C., Cardador M.J., Arce L., Callejón R. (2024). A Comparative Study of Fluorescence and Raman Spectroscopy for Discrimination of Virgin Olive Oil Categories: Chemometric Approaches and Evaluation against Other Techniques. Food Control.

[98] Ballabio D. (2015). A MATLAB Toolbox for Principal Component Analysis and Unsupervised Exploration of Data Structure. Chemom. Intell. Lab. Syst..

[99] Ballabio D., Consonni V. (2013). Classification Tools in Chemistry. Part 1: Linear Models. PLS-DA. Anal. Methods.

[100] Qannari E.M., Wakeling I., MacFie H.J.H. (1995). A Hierarchy of Models for Analysing Sensory Data. Food Qual. Prefer..

[101] Zeaiter M., Rutledge D. (2009). Preprocessing Methods. Comprehensive Chemometrics.

[102] Rocha Baqueta M., Coqueiro A., Henrique Março P., Mandrone M., Poli F., Valderrama P. (2021). Integrated 1H NMR Fingerprint with NIR Spectroscopy, Sensory Properties, and Quality Parameters in a Multi-Block Data Analysis Using ComDim to Evaluate Coffee Blends. Food Chem..

[103] Jouan-Rimbaud Bouveresse D., Pinto R.C., Schmidtke L.M., Locquet N., Rutledge D.N. (2011). Identification of Significant Factors by an Extension of ANOVA–PCA Based on Multi-Block Analysis. Chemom. Intell. Lab. Syst..

[104] Makimori G.Y.F., Bona E. (2019). Commercial Instant Coffee Classification Using an Electronic Nose in Tandem with the ComDim-LDA Approach. Food Anal. Methods.

[105] Galvan D., de Andrade J.C., Conte-Junior C.A., Killner M.H.M., Bona E. (2023). DD-ComDim: A Data-Driven Multiblock Approach for One-Class Classifiers. Chemom. Intell. Lab. Syst..

[106] Mishra P., Roger J.M., Rutledge D.N., Biancolillo A., Marini F., Nordon A., Jouan-Rimbaud-Bouveresse D. (2020). MBA-GUI: A Chemometric Graphical User Interface for Multi-Block Data Visualisation, Regression, Classification, Variable Selection and Automated Pre-Processing. Chemom. Intell. Lab. Syst..

