# Supplementary material for: Geographical Origin Authentication of Leaves and Drupes from Olea europaea via 1H NMR and Excitation–Emission Fluorescence Spectroscopy: A Data Fusion Approach
Source: Molecules. 2025 Jul 30;30(15):3208. doi: 10.3390/molecules30153208 (PMC12348233; doi:10.3390/molecules30153208)
Supplement: Supplementary file 1 [file molecules-30-03208-s001.zip › molecules-3754856-supplementary.pdf]

Supplementary Material

Geographical origin authentication of leaves and drupes from *Olea europaea* via ^{1}H NMR and excitation–emission fluorescence spectroscopy: a data fusion approach

Duccio Tatini[1,2,*], Flavia Bisozzi[1,2], Sara Costantini[1,2], Giacomo Fattori[1,2], Amedeo Boldrini[1,2], Michele Baglioni[1,2,*], Claudia Bonechi[1,2], Alessandro Donati[1,2], Cristiana Tozzi[3], Angelo Riccaboni[3], Gabriella Tamasi[1,2] and Claudio Rossi[1,2]

[1] Department of Biotechnology, Chemistry and Pharmacy, University of Siena, Via Aldo Moro 2, 53100 Siena, Italy
[2] Centre for Colloid and Surface Science (CSGI), University of Florence, Via della Lastruccia 3, 50019 Sesto Fiorentino, Firenze, Italy
[3] Santa Chiara Lab, University of Siena, Via Valdimontone 1, 53100 Siena, Italy
* Correspondence: duccio.tatini@unisi.it (DT), michele.baglioni@unisi.it (MB)

Table S1. Description of the analyzed drupes and leaves samples, including climatic and environmental data.

DRUPES					
**Sample code**	**Geographical area**	**Cultivar**	**Cumulative rainfall (mm)[a]**	**Average Temperature (°C) [a]**	**Altitude (m)**
DR1	Chianti - Siena	Frantoio	439	17.3	262
DR2	Chianti - Siena	Frantoio	439	17.3	262
DR3	Chianti - Siena	Frantoio	439	17.3	262
DR4	Chianti - Siena	Frantoio	550	16.6	304
DR5	Chianti - Siena	Frantoio	550	16.6	300
DR6	Chianti - Siena	Frantoio	550	16.6	300
DR7	Chianti - Siena	Frantoio	550	16.6	279
DR8	Chianti - Siena	Frantoio	550	16.6	286
DR9	Chianti - Siena	Frantoio	519	17.5	305
DR10	Chianti - Siena	Frantoio	519	17.5	296
DR11	Chianti - Siena	Frantoio	519	17.5	296
DR12	Chianti - Siena	Frantoio	616	15.3	292
DR13	Chianti - Siena	Frantoio	616	15.3	292
DR14	Chianti - Siena	Leccino	439	17.3	299
DR15	Chianti - Siena	Leccino	439	17.3	299
DR16	Chianti - Siena	Leccino	439	17.3	262
DR17	Chianti - Siena	Leccino	439	17.3	262
DR18	Chianti - Siena	Leccino	550	16.6	313
DR19	Chianti - Siena	Leccino	550	16.6	313
DR20	Chianti - Siena	Leccino	550	16.6	300
DR21	Chianti - Siena	Leccino	550	16.6	279

DR22	Chianti - Siena	Leccino	550	16.6	286
DR23	Chianti - Siena	Leccino	519	17.5	305
DR24	Chianti - Siena	Leccino	519	17.5	296
DR25	Chianti - Siena	Leccino	519	17.5	296
DR26	Chianti - Siena	Leccino	616	15.3	292
DR27	Chianti - Siena	Leccino	616	15.3	292
DR28	Chianti - Siena	Leccino	616	15.3	268
DR29	Chianti - Siena	Moraiolo	439	17.3	262
DR30	Chianti - Siena	Moraiolo	439	17.3	262
DR31	Chianti - Siena	Moraiolo	550	16.6	300
DR32	Chianti - Siena	Moraiolo	550	16.6	300
DR33	Chianti - Siena	Moraiolo	550	16.6	279
DR34	Chianti - Siena	Moraiolo	550	16.6	286
DR35	Chianti - Siena	Moraiolo	519	17.5	305
DR36	Chianti - Siena	Moraiolo	519	17.5	305
DR37	Chianti - Siena	Moraiolo	616	15.3	292
DR38	Chianti - Siena	Moraiolo	616	15.3	292
DR39	Grosseto	Canino	429	17.0	300
DR40	Grosseto	Frantoio	467	17.3	329
DR41	Grosseto	Frantoio	429	17.0	300
DR42	Grosseto	Leccino	429	17.0	300
DR43	Grosseto	Leccino	319	17.5	217
DR44	Grosseto	Moraiolo	319	17.5	217
DR45	Val d'Orcia	Frantoio	431	15.0	339
DR46	Val d'Orcia	Frantoio	431	15.0	339
DR47	Val d'Orcia	Frantoio	431	15.0	359
DR48	Val d'Orcia	Moraiolo	431	15.0	495
DR49	Val d'Orcia	Moraiolo	431	15.0	495
DR50	Val d'Orcia	Moraiolo	431	15.0	495
DR51	Val d'Orcia	Moraiolo	431	15.0	495
TOTAL (drupes)		51			
			**LEAVES**		
**Sample code**	**Geographical area**	**Cultivar**	**Cumulative rainfall (mm)** [a]	**Average Temperature (°C)** [a]	**Altitude (m)**
FO1	Chianti - Siena	Frantoio	439	17.3	299
FO2	Chianti - Siena	Frantoio	439	17.3	262
FO3	Chianti - Siena	Frantoio	550	16.6	304
FO4	Chianti - Siena	Frantoio	550	16.6	300
FO5	Chianti - Siena	Frantoio	550	16.6	286
FO6	Chianti - Siena	Frantoio	519	17.5	305
FO7	Chianti - Siena	Frantoio	519	17.5	296
FO8	Chianti - Siena	Leccino	616	15.3	292
FO9	Chianti - Siena	Leccino	439	17.3	299
FO10	Chianti - Siena	Leccino	439	17.3	262

FO11	Chianti - Siena	Leccino	550	16.6	313
FO12	Chianti - Siena	Leccino	550	16.6	300
FO13	Chianti - Siena	Leccino	550	16.6	279
FO14	Chianti - Siena	Leccino	519	17.5	305
FO15	Chianti - Siena	Leccino	519	17.5	296
FO16	Chianti - Siena	Leccino	616	15.3	292
FO17	Chianti - Siena	Leccino	616	15.3	268
FO18	Chianti - Siena	Moraiolo	439	17.3	299
FO19	Chianti - Siena	Moraiolo	550	16.6	300
FO20	Chianti - Siena	Moraiolo	550	16.6	286
FO21	Chianti - Siena	Moraiolo	519	17.5	305
FO22	Chianti - Siena	Moraiolo	616	15.3	268
FO23	Grosseto	Canino	429	17.0	300
FO24	Grosseto	Frantoio	429	17.0	300
FO25	Grosseto	Leccino	429	17.0	300
FO26	Grosseto	Leccino	319	17.5	217
FO27	Grosseto	Moraiolo	319	17.5	217
FO28	Val d'Orcia	Frantoio	431	15.0	339
FO29	Val d'Orcia	Leccino	431	15.0	339
FO30	Val d'Orcia	Leccino	431	15.0	359
FO31	Val d'Orcia	Moraiolo	431	15.0	495
TOTAL (leaves)		31			

[a] Cumulative rainfall and average temperatures were calculated between January and September 2022. The data are available here: https://www.sir.toscana.it/consistenza-rete.

Olive leaves ^{1}H-NMR spectroscopy

Table S2. ^{1}H NMR assignment for the olive leaves extracts.

**d (ppm)**	**Assignment**	**Reference**
9.16 – 9.17	Aldehydic proton of olecanthal and its hemiacetal derivative (m)	[1]
9.10 – 9.08	Aldehydic proton of oleacin and its hemiacetal derivative (m)	[1]
8.48	Formate (s)	[2]
7.52	Oleuropein (s)	[3,4]
7.35	Luteolin (m)	[5]
6.88	Luteolin (m)	[5]
6.73	Oleuropein (s)	[3,4]
6.72	Oleuropein (s)	[3,4]
6.69	Oleuropein (d, J = 2 Hz)	[3,4]
6.57	Hydroxytyrosol (dd, J_1= 8.02 Hz, J_2= 1.99 Hz)	[4]
6.52	Fumarate (m)	[2]
6.22	Luteolin (m)	[5]

6.06	Oleuropein (q, J = 6.94 Hz)	[3,4]
5.87	Oleuropein (s)	[3]
5.39	Sucrose anomeric protons (d, J = 3.80 Hz)	[2]
5.13	Anomeric proton of a-glucose (d, J = 3.71 Hz)	[2]
4.52	Anomeric proton of b-glucose (d, J = 7.91 Hz)	[2]
4.16	Oleuropein (m)	[3,4]
3.82	Mannitol (dd, J_1= 11.43 Hz, J_2= 3.32 Hz)	[5,6]
3.77	Mannitol (d, J = 8.11 Hz)	[5,6]
3.73-3.68	Mannitol (m)	[5,6]
3.71	Oleuropein (s)	[3,4]
3.65	Mannitol (dd, J_1= 11.16 Hz, J_2= 6.06 Hz)	[5,6]
3.52 – 3.13	Glucose and sucrose	[3,4]
2.76	Oleuropein (m)	[3,4]
2.70	Malic / Citric acid (d)	[2]
2.67	Oleuropein (d, J = 4.64 Hz)	[3,4]
2.50	Citric acid (d)	[2]
2.42	Oleuropein (d, J = 4.79 Hz)	[3,4]
2.40	Succinic acid (s)	[2]
2.35 – 2.29	Malic acid (m)	[2,5]
2.09 – 1.80	Quinic acid (m)	[6]
1.61	Oleuropein (dd, J_1= 7.01 Hz, J_2= 1.25 Hz)	[3,4]
1.32	Lactic acid (m)	[3,6]
1.13	Maslinic and Oleanolic acid (s)	[7]
0.97	Oleanolic acid (m)	[7]
0.96	Maslinic acid (s)	[7]
0.93	Maslinic and Oleanolic acid (s)	[7]
0.89	Maslinic and Oleanolic acid (s)	[7]
0.80	Maslinic acid (s)	[7]
0.77	Maslinic and Oleanolic acid (s)	[7]

^{1}H-NMR PCA results – Olive leaves

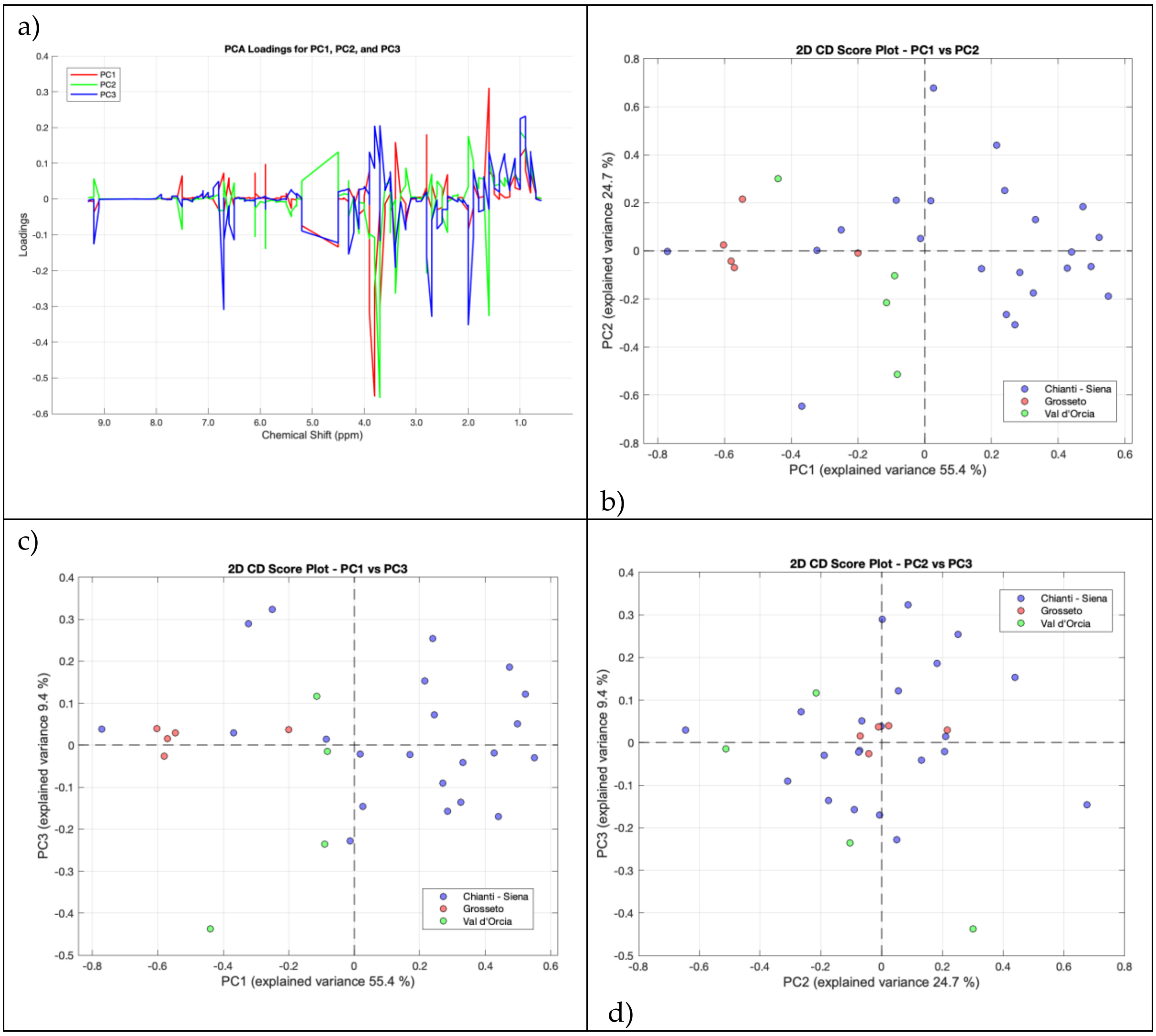

Figure S1. PCA loading plot for ^{1}H NMR data of the olive leaves samples (a) and 2D score plots: PC1 vs PC2 (b), PC1 vs PC3 (c) and PC2 vs PC3 (d).

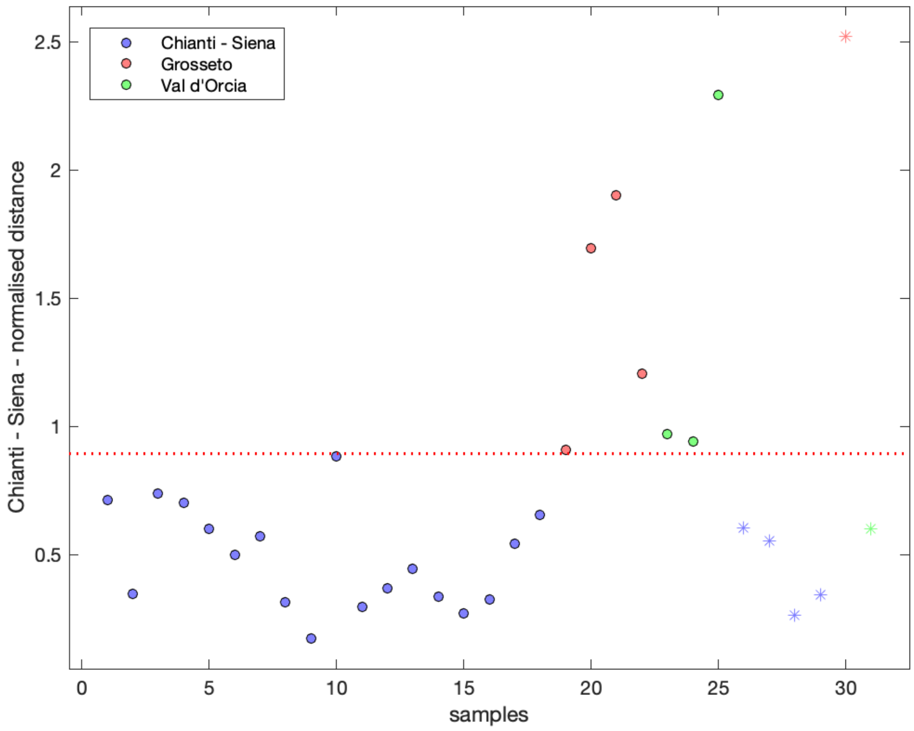

Figure S2. SIMCA normalized distances from ^{1}H NMR data of the olive leaves samples for the modelled Chianti – Siena region.

Olive leaves EEM Fluorescence spectroscopy

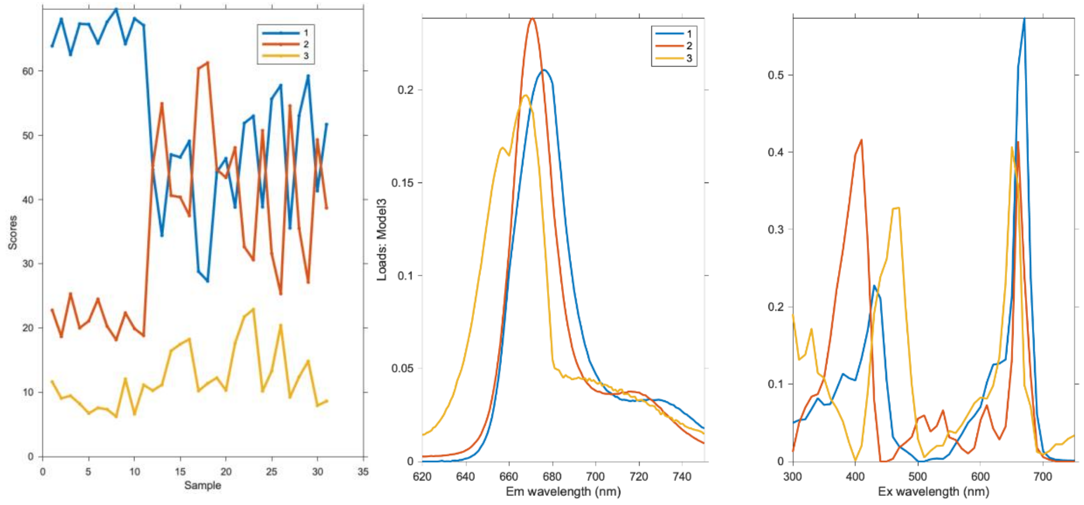

Figure S3. Sample, excitation and emission PARAFAC loadings for the olive leaves samples in spectral Region A.

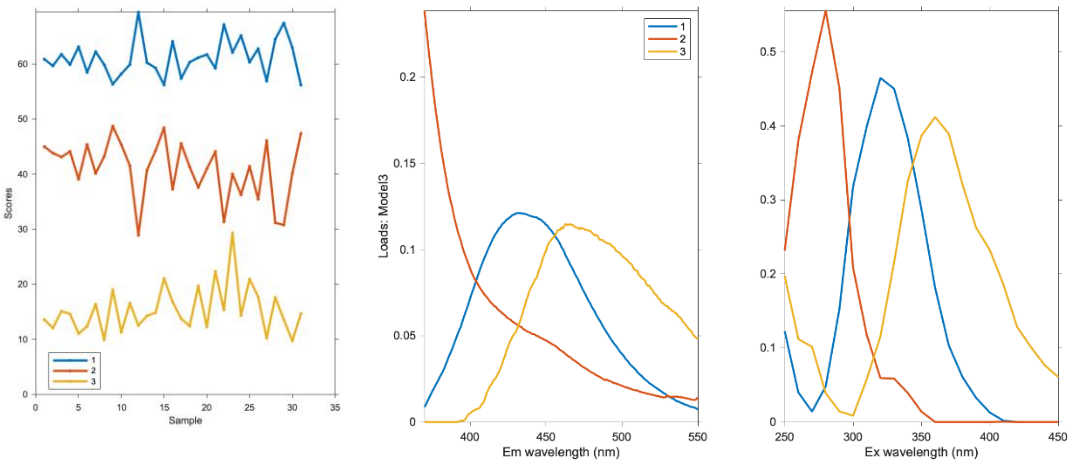

Figure S4. Sample, excitation and emission PARAFAC loadings for the olive leaves samples in spectral Region B.

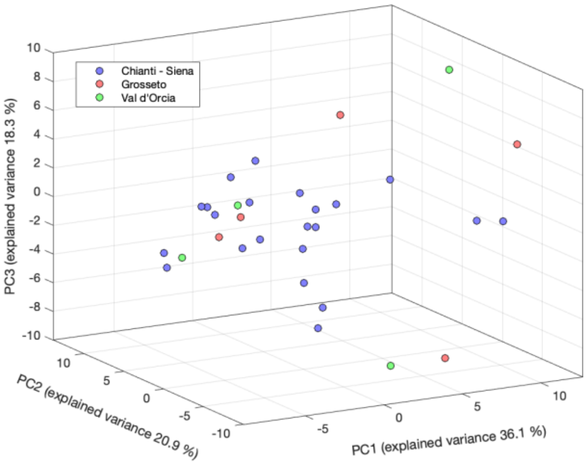

Figure S5. 3D PCA score plot for EEM (Region B) data of the olive leaves samples.

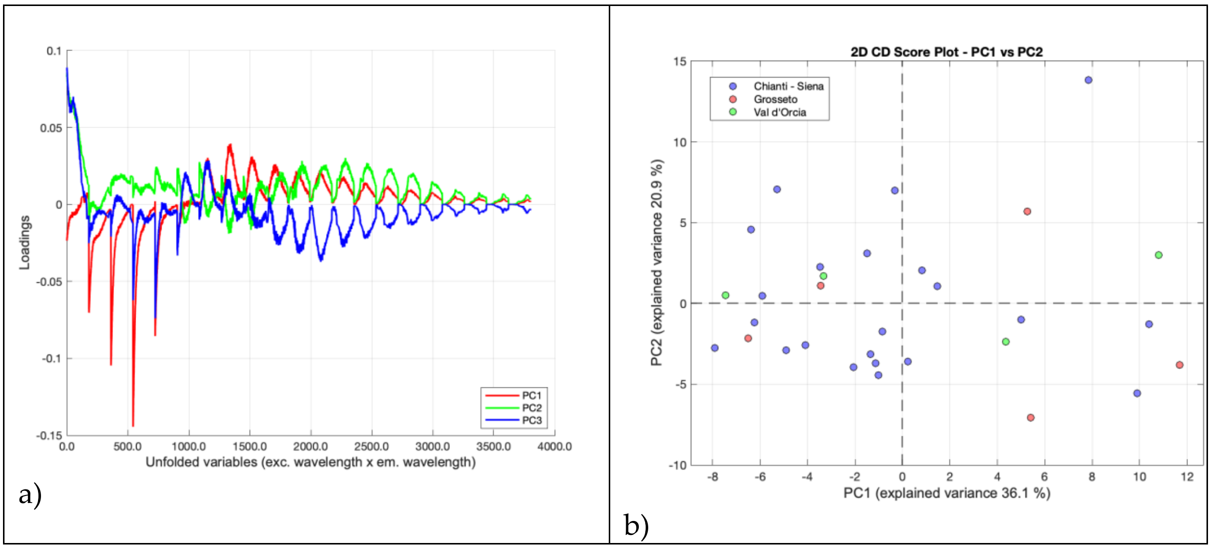

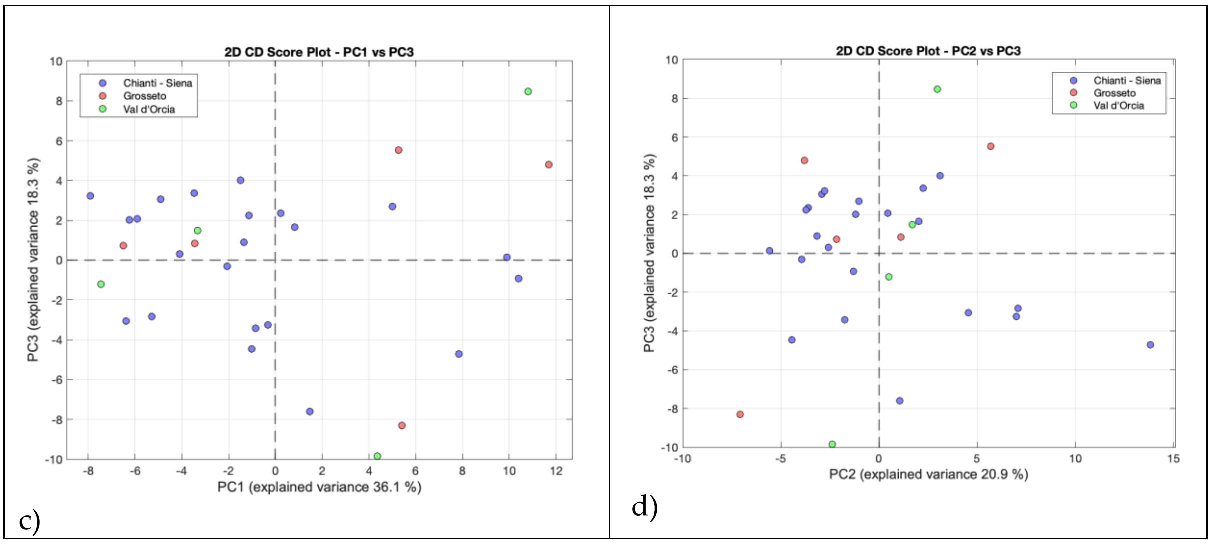

Figure S6. PCA loading plot for EEM (Region B) data of the olive leaves samples (a) and 2D score plots: PC1 vs PC2 (b), PC1 vs PC3 (c) and PC2 vs PC3 (d).

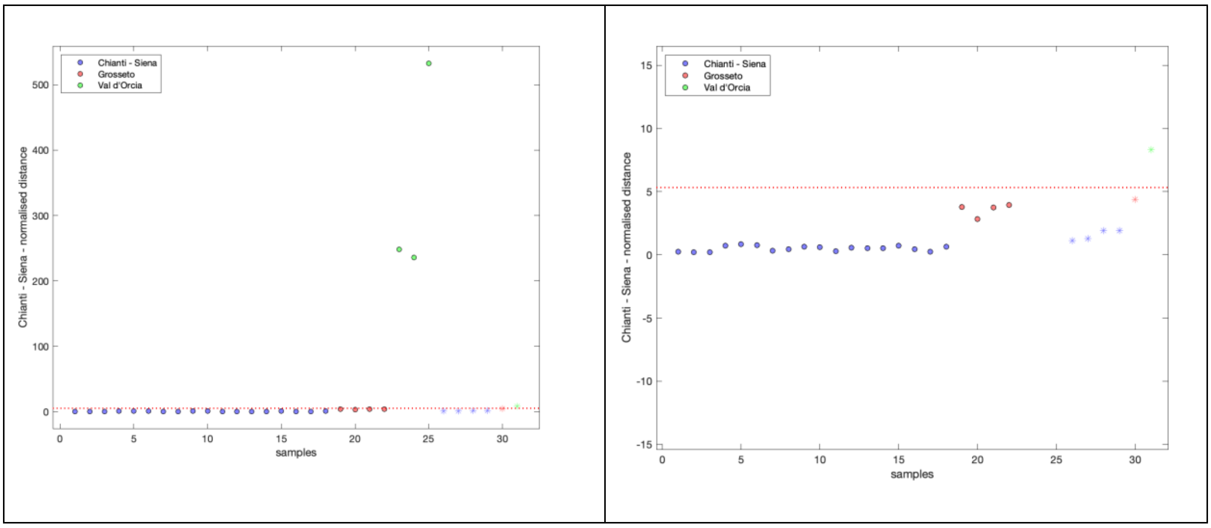

Figure S7. SIMCA normalized distances from EEM data of the olive leaves samples for the modelled Chianti – Siena region. The right panel shows a magnified view of the distance distribution.

Olive Drupes ^{1}H-NMR spectroscopy

Table S3. ^{1}H NMR assignment for the drupes extracts.

d (ppm)	Assignment	Reference
7.68	Quercetin (d, J = 2.2 Hz, H-2′)	[8]
7.54	Verbascoside (m)	[9]
7.52	Oleuropein (s)	[3,4]

7.32	Luteolin (m)	[10]
7.01	Verbascoside (dd, J_1= 8.2 Hz, J_2= 2.0 Hz, H6′′′)	[9]
6.85	Luteolin (d, J = 9.0 Hz, H-5′)	[10]
6.74 – 6.78	DHPEA-EDA (dihydroxyphenylethanolelenolic acid dialdehyde)/Oleocanthal/Oleomissional (m)	[11]
6.73	Oleuropein (s)	[3,4]
6.71	Oleuropein (d, J = 2 Hz)	[3,4]
6.63	Verbascoside (m)	[9]
6.61	Verbascoside (m)	[9]
6.59	Hydroxytyrosol (d, J = 2 Hz)	[12]
6.31	Verbascoside (d, J = 15.8 Hz)	[9]
6.07	Oleuropein (q, J = 6.94 Hz)	[3,4]
5.89	Oleuropein (s)	[3]
5.19 – 5.22	Maslinic and oleanolic acid (m)	[13]
5.15	Verbascoside (m)	[9]
4.52	Anomeric proton of b-glucose (d, J = 7.91 Hz)	[2]
4.07	Lactic acid (m)	[14]
3.83	Mannitol (dd, J_1= 11.43 Hz, J_2= 3.32 Hz)	[5,6]
3.79	Mannitol (d, J = 8.11 Hz)	[5,6]
3.73-3.68	Mannitol (m)	[5,6]
3.65	Mannitol (dd, J_1= 11.16 Hz, J_2= 6.06 Hz)	[5,6]
3.54 – 3.14	Glucose and sucrose	[3,4]
2.84	Maslinic and oleanolic acid (dd, J_1= 14.0 Hz, J_2= 4.1 Hz)	[13]
2.76	Oleuropein (m)	[3,4]
2.69	Oleuropein (d, J = 4.64 Hz)	[3,4]
2.64	Tyrosol (m)	[14]
2.61	Hydroxytyrosol (m)	[12]
2.40	Succinic acid (s)	[3]
2.36 – 2.31	Malic acid (m)	[3,5]
1.95 – 1.86	Quinic acid (m)	[6]
1.85	Acetic acid (s)	[9]
1.61	Oleuropein (m)	[3,4]
1.32	Lactic acid (m)	[3,6]
1.13	Maslinic and Oleanolic acid (s)	[7]
1.07	Verbascoside (d, J = 6.3 Hz)	[9]
0.97	Oleanolic acid (m)	[7]
0.96	Maslinic acid (s)	[7]
0.93	Maslinic and oleanolic acid (s)	[7]
0.89	Maslinic and oleanolic acid (s)	[7]
0.81	Maslinic acid (s) and oleanolic acid (s)	[7]
0.77	Maslinic and oleanolic acid (s)	[7]

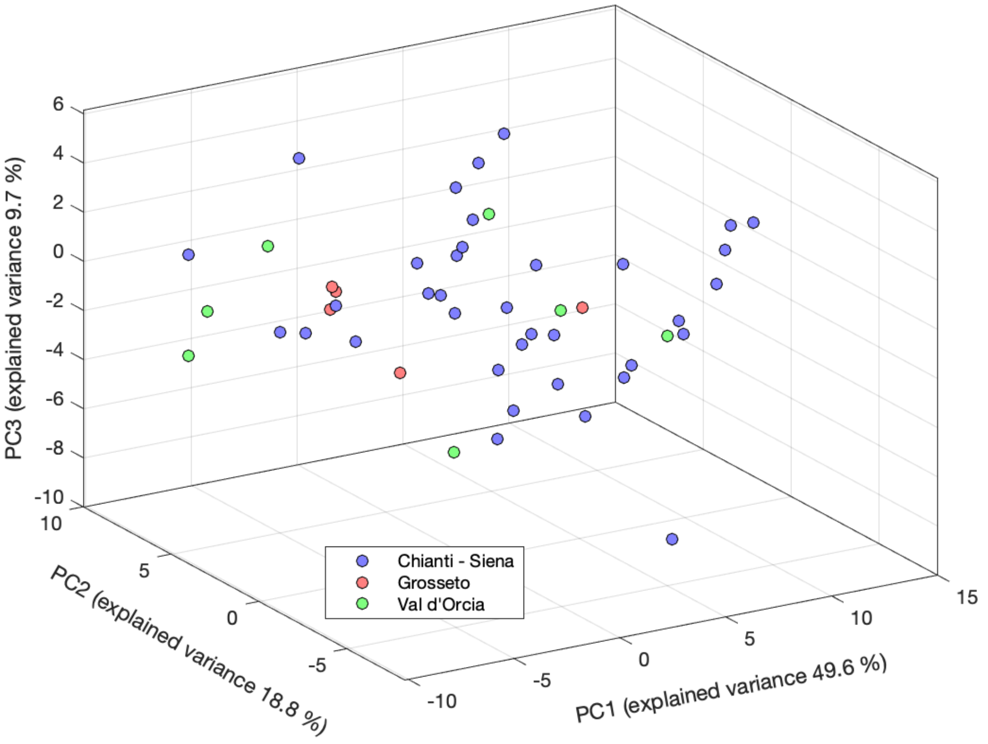

Figure S8. 3D PCA score plot for ^{1}H NMR data of the olive drupes samples.

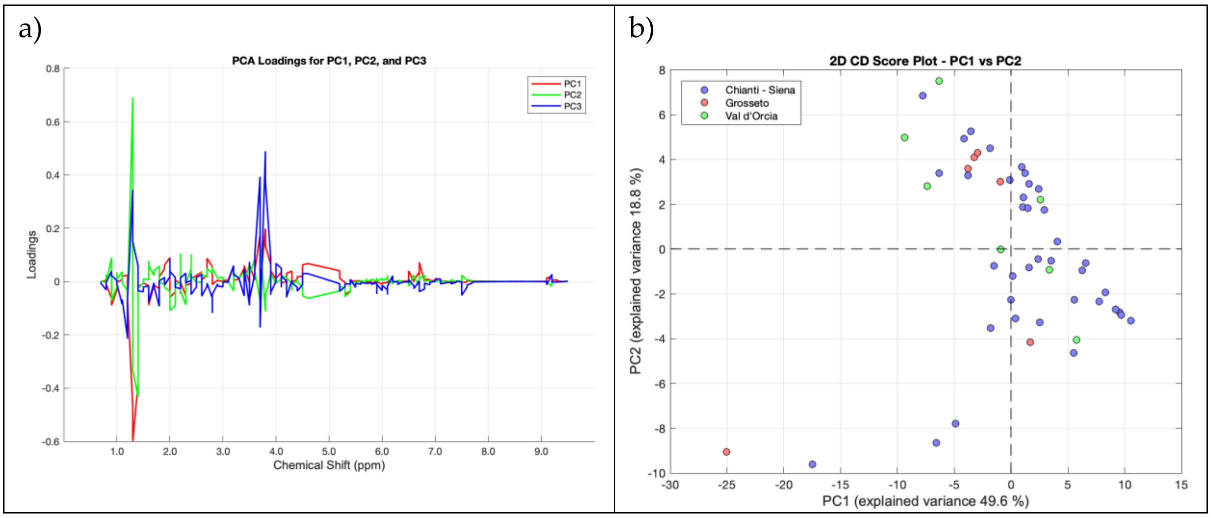

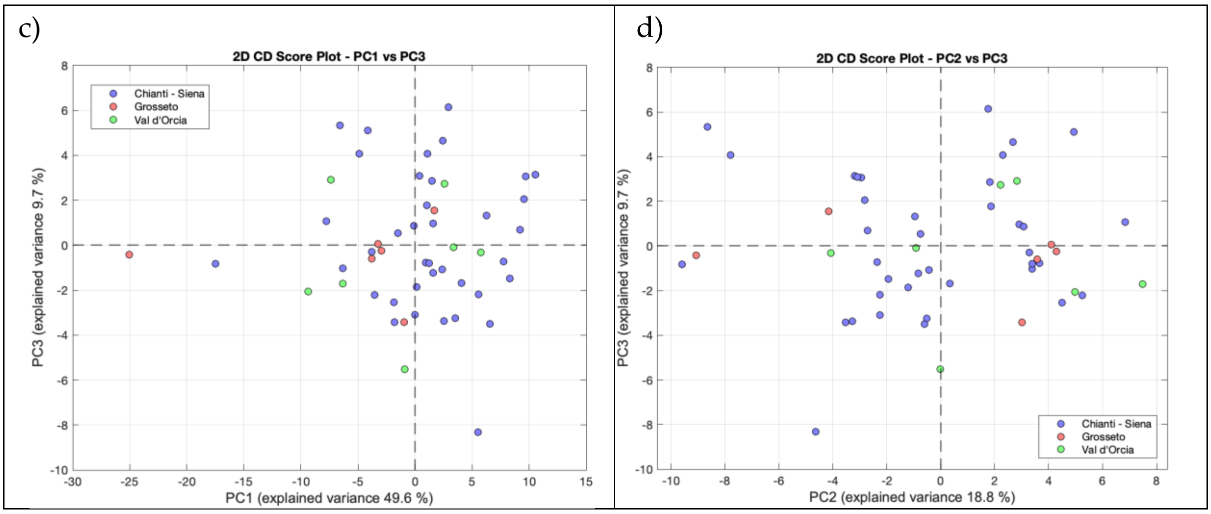

Figure S9. PCA loading plot for ^{1}H NMR data of the olive drupes samples (a) and 2D score plots: PC1 vs PC2 (b), PC1 vs PC3 (c) and PC2 vs PC3 (d).

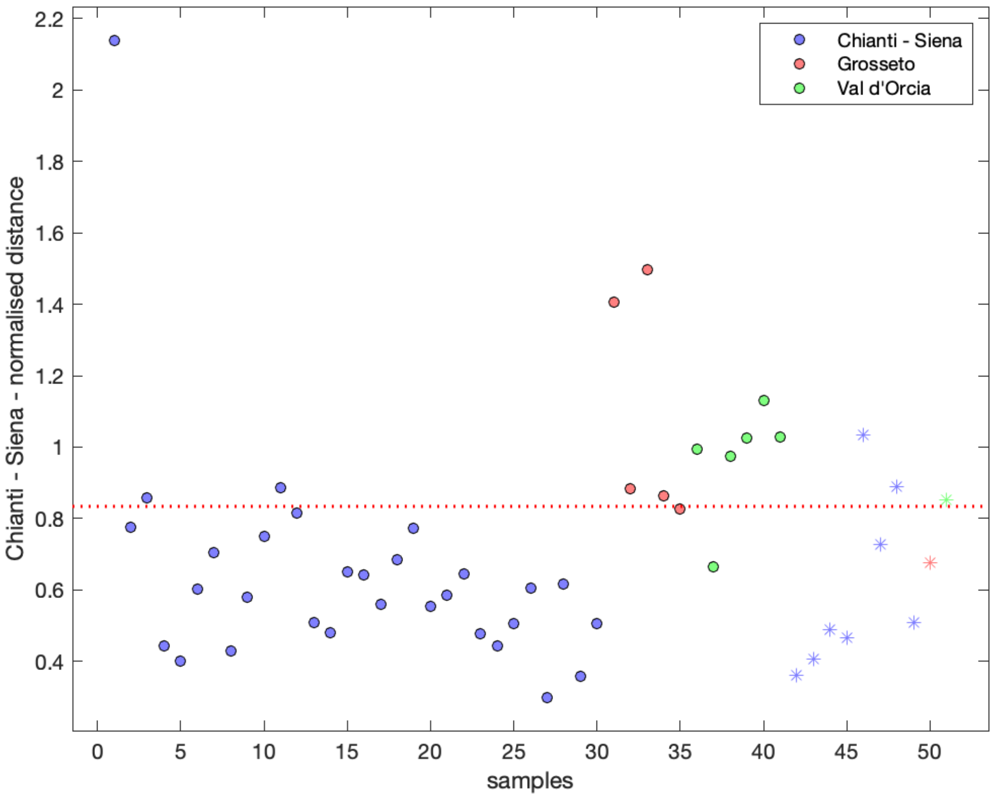

Figure S10. SIMCA normalized distances from EEM data of the olive drupes samples for the modelled Chianti – Siena region.

Olive Drupes EEM Fluorescence spectroscopy

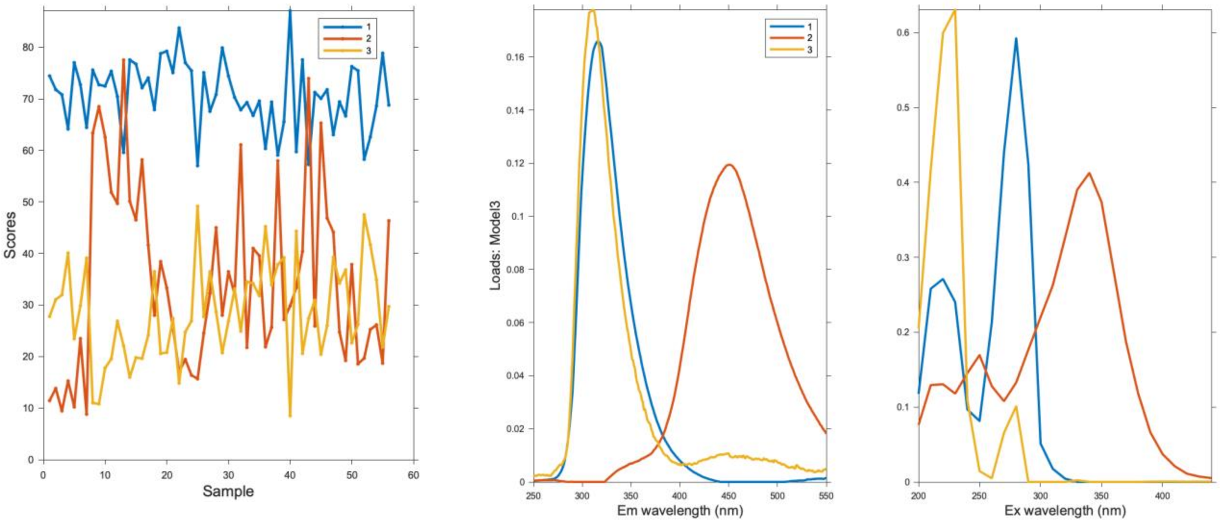

Figure S11. Sample, excitation and emission PARAFAC loadings for the drupe samples.

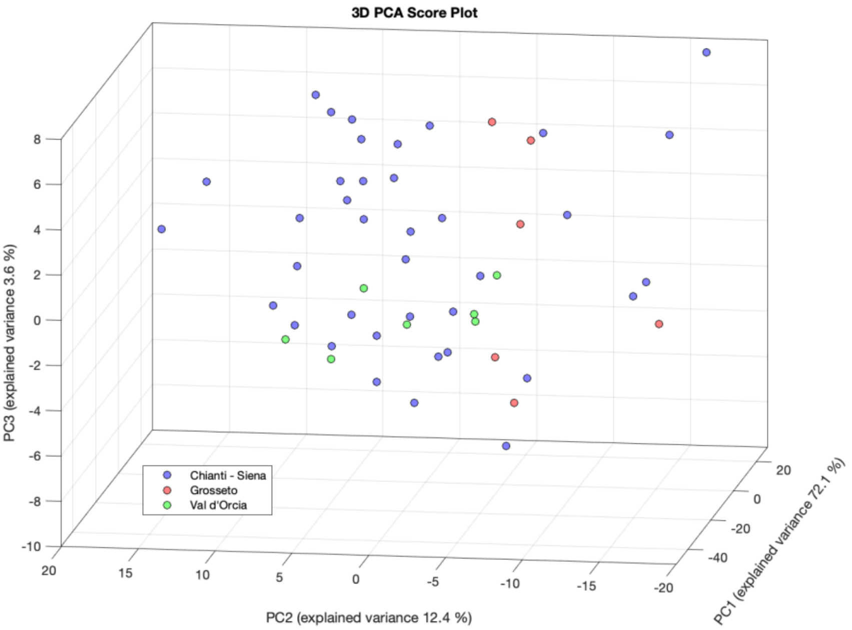

Figure S12. 3D PCA score plot for EEM data of the olive drupes samples.

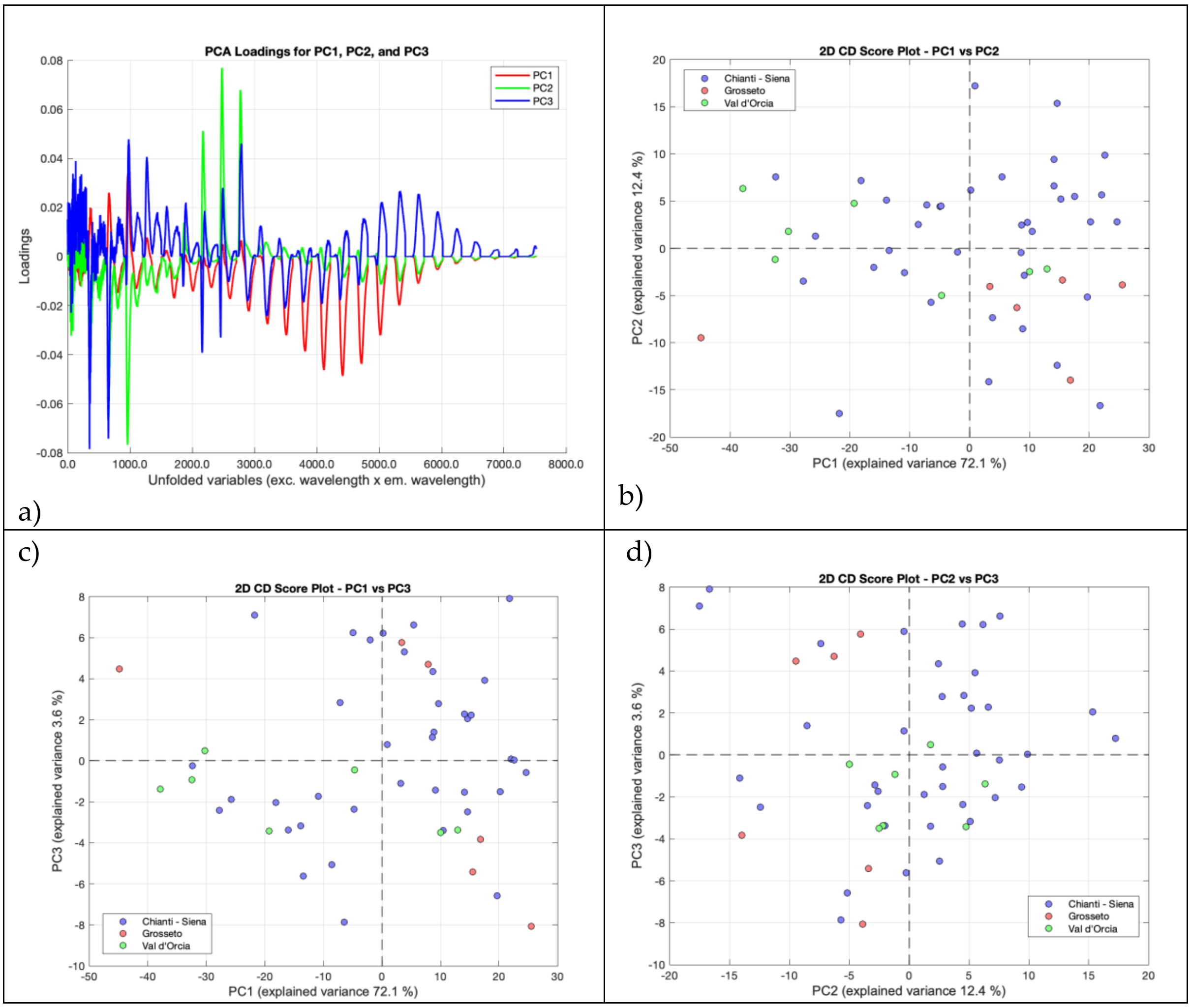

Figure S13. PCA loading plot for EEM data of the olive drupes samples (a) and 2D score plots: PC1 vs PC2 (b), PC1 vs PC3 (c) and PC2 vs PC3 (d).

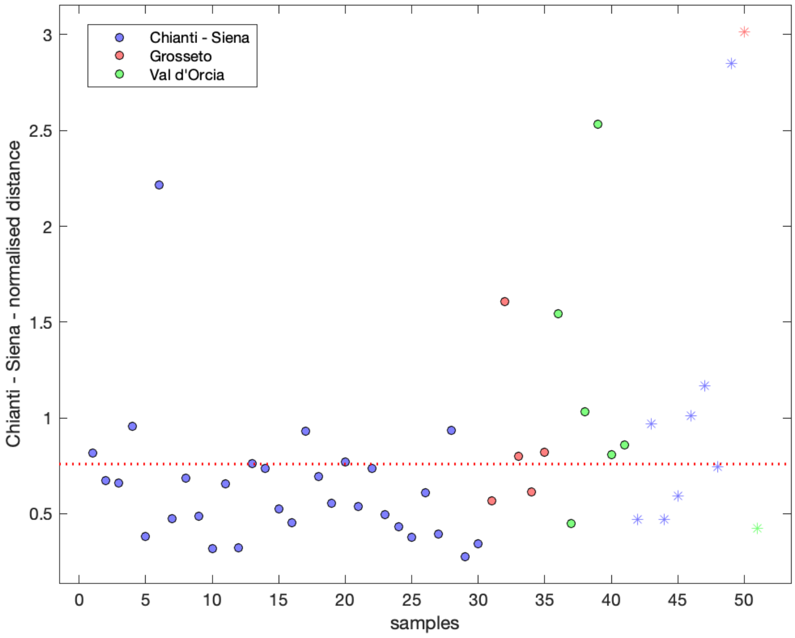

Figure S14. SIMCA normalized distances from EEM data of the olive drupes samples for the modelled Chianti – Siena region.

Mid-level data fusion (ComDim) results – Olive leaves and drupes

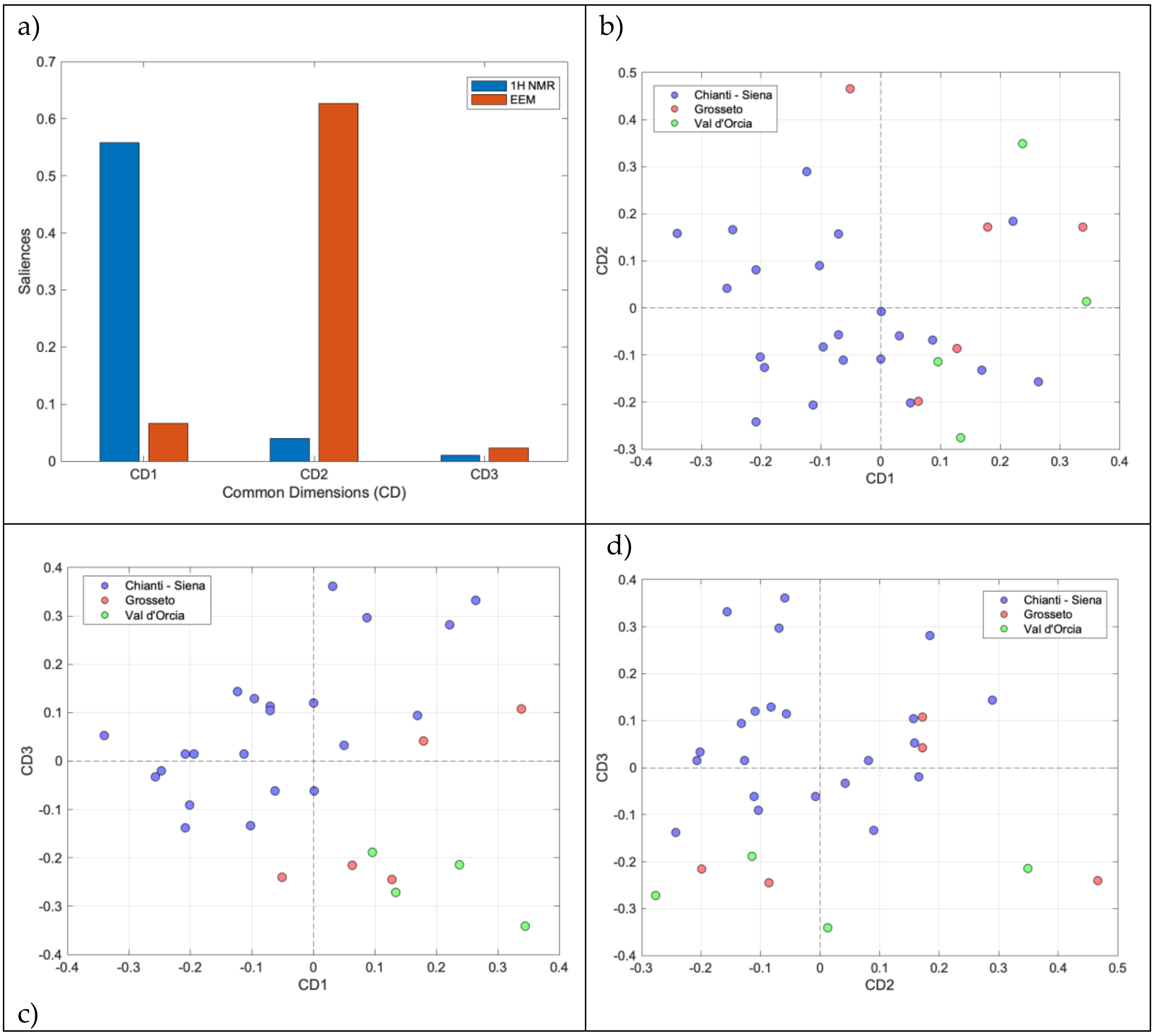

Figure S15. ComDim salience plot for the leaves samples (a) and 2D ComDim score plots: CD1 vs CD2 (b), CD1 vs CD3 (c) and CD2 vs CD3 (d).

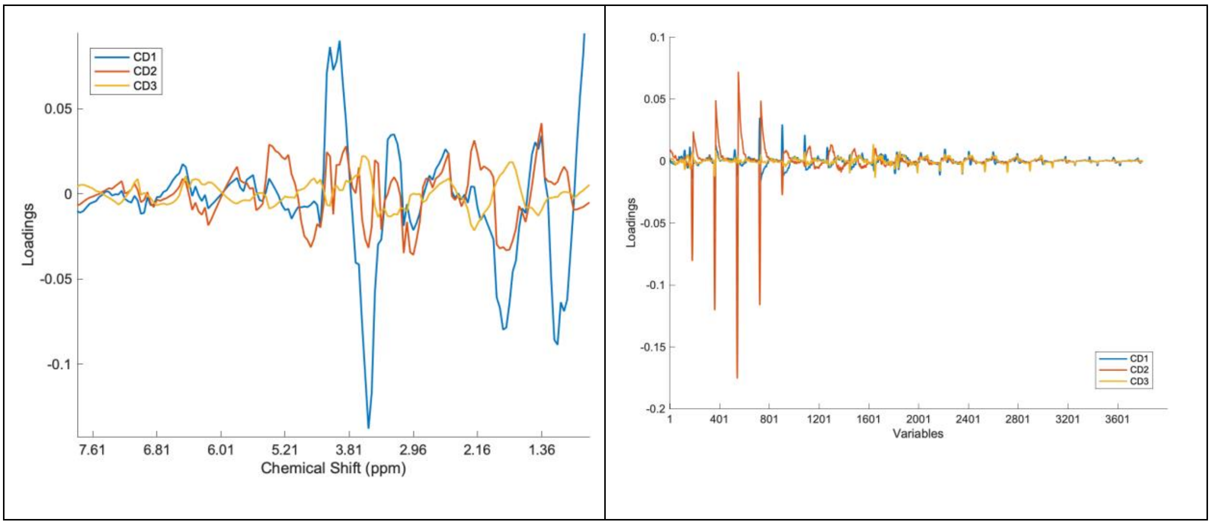

Figure S16. ComDim loadings for the ^{1}H NMR (left panel) and EEM block (right panel) for each Common Dimension calculated for the olive leaves dataset. The EEM loadings are reported as a function of the folded original variables, i.e. excitation x emission wavelengths.

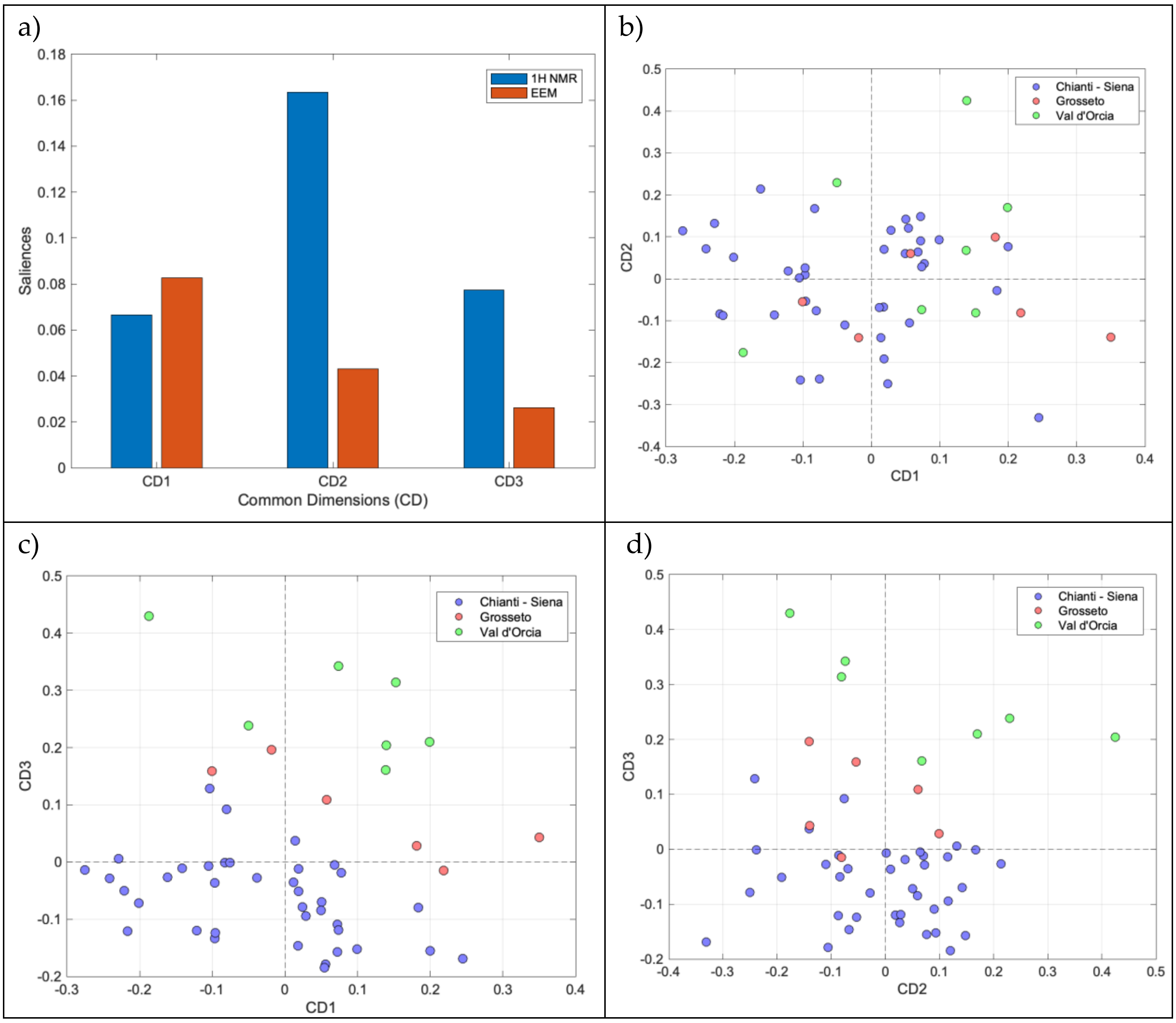

Figure S17. ComDim salience plot for the drupes samples (a) and 2D ComDim score plots: CD1 vs CD2 (b), CD1 vs CD3 (c) and CD2 vs CD3 (d).

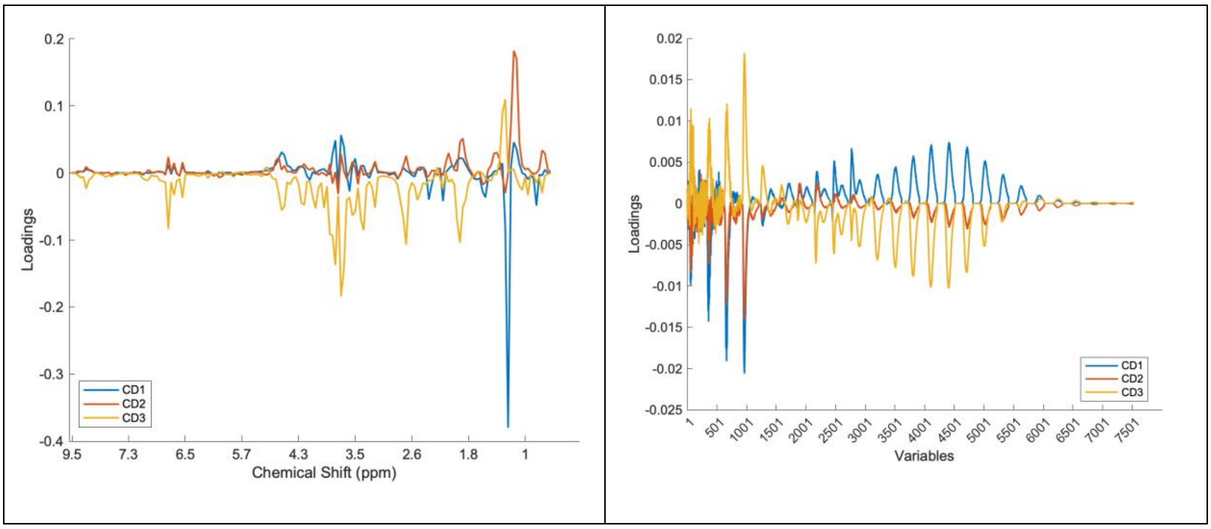

Figure S18. ComDim loadings for the ^{1}H NMR (left panel) and EEM block (right panel) for each Common Dimension calculated for the olive drupes dataset. The EEM loadings are reported as a function of the folded original variables, i.e. excitation x emission wavelengths.

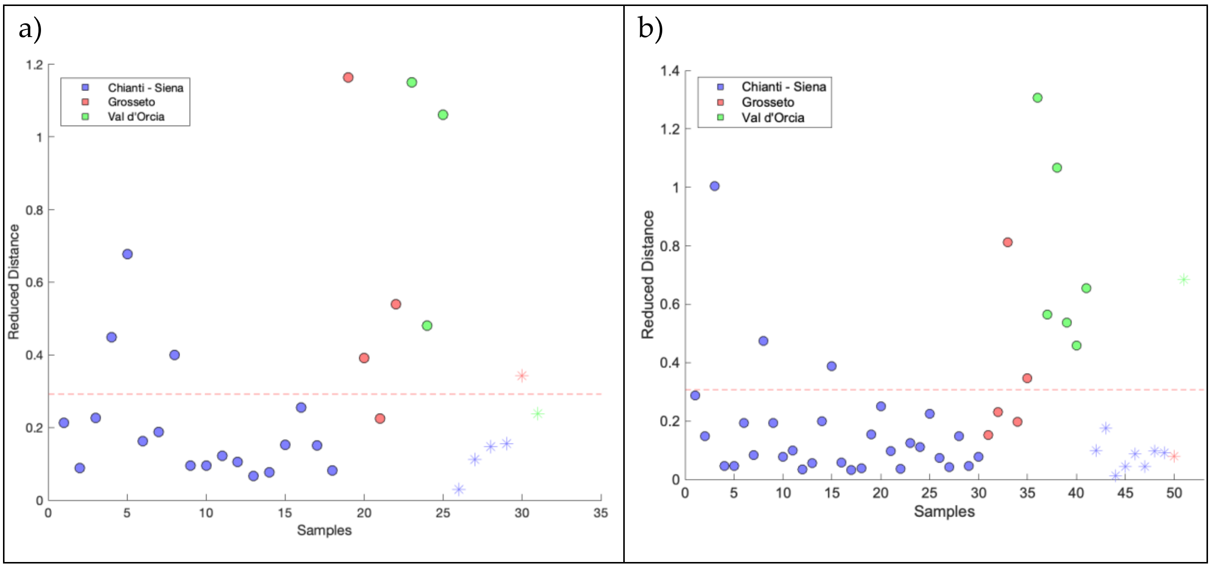

Figure S19. Reduced distances calculated from the ComDim-based SIMCA multiblock model for the leaves (a) and drupes (b) drupes samples for the Chianti – Siena region.

References

1. Karkoula, E.; Skantzari, A.; Melliou, E.; Magiatis, P. Direct Measurement of Oleocanthal and Oleacein Levels in Olive Oil by Quantitative[1] H NMR. Establishment of a New Index

for the Characterization of Extra Virgin Olive Oils. *J. Agric. Food Chem.* **2012**, *60*, 11696–11703, doi:10.1021/jf3032765.

2. Girelli, C.R.; Angilè, F.; Del Coco, L.; Migoni, D.; Zampella, L.; Marcelletti, S.; Cristella, N.; Marangi, P.; Scortichini, M.; Fanizzi, F.P. 1H-NMR Metabolite Fingerprinting Analysis Reveals a Disease Biomarker and a Field Treatment Response in Xylella Fastidiosa Subsp. Pauca-Infected Olive Trees. *Plants* **2019**, *8*, 115, doi:10.3390/plants8050115.
3. Huertas-Alonso, A.J.; Gavahian, M.; González-Serrano, D.J.; Hadidi, M.; Salgado-Ramos, M.; Sánchez-Verdú, M.P.; Simirgiotis, M.J.; Barba, F.J.; Franco, D.; Lorenzo, J.M.; et al. Valorization of Wastewater from Table Olives: NMR Identification of Antioxidant Phenolic Fraction and Microwave Single-Phase Reaction of Sugary Fraction. *Antioxidants* **2021**, *10*, 1652, doi:10.3390/antiox10111652.
4. Goulas, V.; Exarchou, V.; Troganis, A.N.; Psomiadou, E.; Fotsis, T.; Briasoulis, E.; Gerothanassis, I.P. Phytochemicals in Olive-leaf Extracts and Their Antiproliferative Activity against Cancer and Endothelial Cells. *Molecular Nutrition Food Res* **2009**, *53*, 600–608, doi:10.1002/mnfr.200800204.
5. Wishart, D.S.; Knox, C.; Guo, A.C.; Eisner, R.; Young, N.; Gautam, B.; Hau, D.D.; Psychogios, N.; Dong, E.; Bouatra, S.; et al. HMDB: A Knowledgebase for the Human Metabolome. *Nucleic Acids Research* **2009**, *37*, D603–D610, doi:10.1093/nar/gkn810.
6. Girelli, C.R.; Hussain, M.; Verweire, D.; Oehl, M.C.; Massana-Codina, J.; Avendaño, M.S.; Migoni, D.; Scortichini, M.; Fanizzi, F.P. Agro-Active Endo-Therapy Treated Xylella Fastidiosa Subsp. Pauca-Infected Olive Trees Assessed by the First 1H-NMR-Based Metabolomic Study. *Sci Rep* **2022**, *12*, 5973, doi:10.1038/s41598-022-09687-8.
7. Agatonovic-Kustrin, S.; Gegechkori, V.; Morton, D.W.; Tucci, J.; Mohammed, E.U.R.; Ku, H. The Bioprofiling of Antibacterials in Olive Leaf Extracts via Thin Layer Chromatography-Effect Directed Analysis (TLC-EDA). *Journal of Pharmaceutical and Biomedical Analysis* **2022**, *219*, 114916, doi:10.1016/j.jpba.2022.114916.
8. Tasnuva, S.T.; Qamar, U.A.; Ghafoor, K.; Sahena, F.; Jahurul, M.H.A.; Rukshana, A.H.; Juliana, M.J.; Al-Juhaimi, F.Y.; Jalifah, L.; Jalal, K.C.A.; et al. α-Glucosidase Inhibitors Isolated from *Mimosa Pudica* L. *Natural Product Research* **2019**, *33*, 1495–1499, doi:10.1080/14786419.2017.1419224.
9. Beteinakis, S.; Papachristodoulou, A.; Gogou, G.; Katsikis, S.; Mikros, E.; Halabalaki, M. NMR-Based Metabolic Profiling of Edible Olives—Determination of Quality Parameters. *Molecules* **2020**, *25*, 3339, doi:10.3390/molecules25153339.
10. Wang, L.; Li, X.; Zhang, S.; Lu, W.; Liao, S.; Liu, X.; Shan, L.; Shen, X.; Jiang, H.; Zhang, W.; et al. Natural Products as a Gold Mine for Selective Matrix Metalloproteinases Inhibitors. *Bioorganic & Medicinal Chemistry* **2012**, *20*, 4164–4171, doi:10.1016/j.bmc.2012.04.063.
11. Esposito, A.; De Luca, P.F.; Graziani, V.; D'Abrosca, B.; Fiorentino, A.; Scognamiglio, M. Phytochemical Characterization of Olea Europaea L. Cultivars of Cilento National Park (South Italy) through NMR-Based Metabolomics. *Molecules* **2021**, *26*, 3845, doi:10.3390/molecules26133845.
12. Kalampaliki, A.D.; Giannouli, V.; Skaltsounis, A.-L.; Kostakis, I.K. A Three-Step, Gram-Scale Synthesis of Hydroxytyrosol, Hydroxytyrosol Acetate, and 3,4-Dihydroxyphenylglycol. *Molecules* **2019**, *24*, 3239, doi:10.3390/molecules24183239.
13. De Cássia Lemos Lima, R.; T. Kongstad, K.; Kato, L.; José das Silva, M.; Franzyk, H.; Staerk, D. High-Resolution PTP1B Inhibition Profiling Combined with HPLC-HRMS-SPE-NMR for Identification of PTP1B Inhibitors from Miconia Albicans. *Molecules* **2018**, *23*, 1755, doi:10.3390/molecules23071755.

14. Beteinakis, S.; Papachristodoulou, A.; Stathopoulos, P.; Mikros, E.; Halabalaki, M. A Multilevel LC-HRMS and NMR Correlation Workflow towards Foodomics Advancement: Application in Table Olives. *Talanta* **2024**, *280*, 126641, doi:10.1016/j.talanta.2024.126641.
